# Predicting delirium in critically Ill COVID-19 patients using EEG-derived data: a machine learning approach

**DOI:** 10.1007/s11357-025-01809-0

**Published:** 2025-07-23

**Authors:** Ana Viegas, Cristiana P. Von Rekowski, Rúben Araújo, Luís Ramalhete, Inês Menezes Cordeiro, Manuel Manita, Miguel Viana-Baptista, Paula Macedo, Luís Bento

**Affiliations:** 1 NMS – NOVA Medical School, FCM – Faculdade de Ciências Médicas, Universidade NOVA de Lisboa, Campo Dos Mártires da Pátria 130, 1169-056 Lisbon, Portugal; 2 CHRC – Comprehensive Health Research Centre, Universidade NOVA de Lisboa, Campo Dos Mártires da Pátria 130, 1150-082 Lisbon, Portugal; 3 ESTeSL – Escola Superior de Tecnologia da Saúde de Lisboa, Instituto Politécnico de Lisboa, Avenida D. João II, Lote 4.69.01, Parque das Nações, 1990-096 Lisbon, Portugal; 4 H&TRC – Health & Technology Research Center, ESTeSL – Escola Superior de Tecnologia da Saúde de Lisboa, Instituto Politécnico de Lisboa, Avenida D. João II, Lote 4.69.01, Parque das Nações, 1990-096 Lisbon, Portugal; 5 Neurosciences Area, Clinical Neurophysiology Unit, ULSSJ – Unidade Local de Saúde São José, Rua José António Serrano, 1150-199 Lisbon, Portugal; 6 ISEL– Instituto Superior de Engenharia de Lisboa, Instituto Politécnico de Lisboa, Rua Conselheiro Emídio Navarro 1, 1959-007 Lisbon, Portugal; 7 Blood and Transplantation Center of Lisbon, Instituto Português Do Sangue E da Transplantação, Avenida Miguel Bombarda 6, 1000-208 Lisbon, Portugal; 8 iNOVA4Health – Advancing Precision Medicine, NOVA Medical School, FCM – Faculdade de Ciências Médicas, Universidade NOVA de Lisboa, Campo Dos Mártires da Pátria 130, 1169-056 Lisbon, Portugal; 9 Neurology Department, ULSSJ – Unidade Local de Saúde São José, Rua José António Serrano, 1150-199 Lisbon, Portugal; 10 Intensive Care Department, ULSSJ – Unidade Local de Saúde São José, Rua José António Serrano, 1150-199 Lisbon, Portugal; 11 Neurology Department, ULSLO – Unidade Local de Saúde de Lisboa Ocidental, Rua da Junqueira 126, 1349-019 Lisbon, Portugal; 12 CCAL – Centro Clínico Académico de Lisboa, NOVA Medical School, FCM – Faculdade de Ciências Médicas, Universidade NOVA de Lisboa, Campo Dos Mártires da Pátria 130, 1169-056 Lisbon, Portugal

**Keywords:** Delirium, EEG, COVID-19, SARS-CoV-2 infection, ICU, Machine learning

## Abstract

Delirium is a severe and common complication among critically ill patients, particularly those with SARS-CoV-2 infection, contributing to increased morbidity and mortality. Early identification of at-risk patients is crucial for timely intervention and improved outcomes. This prospective observational cohort study explores the potential of electroencephalography (EEG) combined with machine learning (ML) models for predicting delirium in critically ill patients with SARS-CoV-2 infection. A stepwise modeling approach was applied, starting with the independent analysis of specific EEG variables to assess their predictive value. Subsequently, three ML models were developed using data from 70 patients (31 with delirium, 39 without): two relied solely on EEG data, while the third integrated demographic, clinical, laboratory, and EEG data. An additional model analyzed EEG data before and after delirium diagnosis in 11 patients. Several EEG features were identified as predictors of delirium, with increased theta activity emerging as the most consistent. The best EEG-only model achieved an area under the curve (AUC) of 0.733 (sensitivity = 0.645, specificity = 0.692), indicating moderate predictive performance. Including demographic, clinical, and laboratory variables improved performance (AUC = 0.825, sensitivity = 0.613, specificity = 0.795). The model analyzing EEG features before and after delirium diagnosis achieved the highest accuracy (AUC = 0.950, sensitivity and specificity = 0.818), reinforcing the value of EEG-based monitoring. EEG-based ML models show promise for predicting delirium in critically ill patients, with increased theta activity identified as a key predictor. However, their moderate AUC, sensitivity, and specificity highlight the need for further refinement.

**Graphical Abstract:**

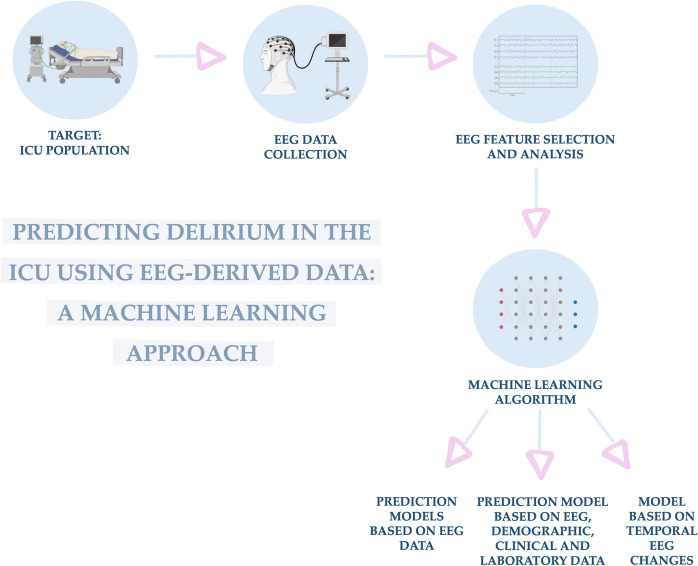

**Supplementary Information:**

The online version contains supplementary material available at 10.1007/s11357-025-01809-0.

## Introduction

Delirium, an acute confusional state characterized by fluctuating disturbances in attention and cognition, is a common and severe complication among critically ill patients, particularly those with severe acute respiratory syndrome due to coronavirus 2 (SARS-CoV-2) infection [1–3]. The incidence of delirium in patients with severe COVID-19 is remarkably high, with rates reaching up to 80% in the most critically ill patients [4–8], further complicating their recovery and exacerbating their already fragile state [8, 9]. Contributing factors include severe respiratory failure, prolonged Invasive Mechanical Ventilation (IMV) with deep sedation, isolation, and the systemic inflammatory response associated with COVID-19 [3, 10–12]. Consequently, delirium not only prolongs hospital stays but also significantly increases morbidity and mortality rates, making it a paramount concern for healthcare providers [8, 13–15]. The immense strain of delirium on healthcare systems is evident in the increased need for intensive care resources and extended hospital stays, which substantially elevate healthcare costs [16–18]. Despite being a well-recognized complication of critical illness and COVID-19, delirium often remains under-recognized and poorly managed, underscoring the critical need for improved detection and management strategies to better support these vulnerable patients [19–21].

Early identification of patients at risk can facilitate timely interventions, potentially mitigating adverse outcomes and improving the overall prognosis [22–24]. By anticipating such events, healthcare practitioners can implement strategies to manage and possibly prevent their occurrence, thereby reducing the burden on healthcare systems [25, 26]. However, current detection and prediction methods, such as clinical assessments using the Confusion Assessment Method for the Intensive Care Unit (CAM-ICU) [27], often fail to identify delirium early, leading to delayed interventions [28]. Additionally, in resource-constrained Intensive Care Unit (ICU) environments, especially under adverse events, such as the COVID-19 pandemic, the frequency of delirium assessments often decreased because of revised diagnostic and treatment protocols, which inadvertently increased the risk, severity, and duration of delirium, leading to higher rates of adverse outcomes [29–35].

Electroencephalography (EEG) is a non-invasive and effective method for monitoring brain activity and has been identified as a valuable tool not only for detecting delirium [36–41] but also for predicting its onset, as it captures changes in EEG waveforms that occur prior to observable changes in an individual’s behavior [42, 43]. Similar to the use of resting-state EEG to assess baseline brain health and neurocognitive vulnerability in patients with neurodegenerative diseases, this technique can serve as a valuable tool for detecting early cerebral dysregulation in critically ill ICU patients before the overt symptoms of delirium appear [44]. Although the utility of EEG in delirium was established long ago [45], the temporal patterns of EEG signatures associated with ICU delirium remain poorly characterized [46]. Nevertheless, EEG biomarkers, such as changes in brain wave frequency and pattern, have been linked to delirium, providing critical insights into the neural underpinnings of this condition. These biomarkers include increases in delta and theta power, decreases in alpha power, and disruptions in beta rhythms, which may signal the occurrence of delirium even before the clinical signs appear [40, 47, 48]. Additionally, frontal lobe abnormalities detected through EEG, such as slow waves, background rhythm slowing, and epileptiform discharges, have been proposed as potential EEG indicators of COVID-19-associated encephalopathy and delirium [34, 49]. The selective vulnerability of frontal lobe circuits to SARS-CoV-2 infection, potentially leading to neuropsychiatric symptoms including delirium, has also been emphasized [49]. Acute encephalopathy presenting as delirium has also been characterized by slow arrhythmic waves with the suppression of alpha activity, which is known as polymorphic delta activity (PDA) [50]. Similarly, specific EEG abnormalities—including the absence of reactivity, a delta-predominant background, and periodic discharges (PDs)—have been recognized as independent predictors of ICU mortality and are associated with delirium in septic ICU patients [51]. Moreover, burst-suppression patterns have also been observed as markers of severe brain dysfunction in delirious patients [41, 52–55], and the relationship between EEG suppression and delirium has been explored in older adults undergoing major surgery, in whom a suppressed EEG pattern indicative of deep anesthesia is linked to a higher incidence of postoperative delirium and mortality, highlighting the clinical relevance of EEG monitoring in delirium management [56]. Based on the findings from a systematic review, EEG abnormalities, such as increased burst-suppression, early EEG slowing (increased delta and theta power), electrographic seizures, and reduced functional connectivity, are considered potential precursors to the development of delirium, highlighting their value as early indicators in at-risk patients [47]. However, research on pre-delirium EEG findings is limited, and the existing studies are constrained by small sample sizes, a predominant focus on postoperative delirium rather than ICU delirium, and a lack of standardized EEG protocols. These limitations restrict the generalizability of the results and hinder the development of prospective studies in broader and more diverse populations, thereby complicating the interpretation and application of the findings across different clinical settings.

Leveraging the power of machine learning (ML), researchers have developed models that use EEG data to predict or detect delirium in critically ill patients [48, 57]. The advantage of these ML algorithms lies in their ability to analyze complex EEG patterns and identify subtle changes that may indicate impending delirium [58]. For instance, a predictive model for postoperative delirium in elderly patients undergoing major elective non-cardiac surgery was developed using ML techniques [59]. The model, which combined the burst-suppression ratio, spectral features, and covariance matrices with the clinical data, achieved an AUC of 0.77. Notably, when evaluating specific anesthetic agents, the AUC reached 0.80 for patients receiving Sevoflurane, a volatile anesthetic. ML algorithms have also been applied to preoperative EEG data to successfully predict postoperative delirium after cardiovascular surgeries in 128 patients, achieving an accuracy of 86% and an AUC of 0.93 [60]. The feasibility of predicting delirium from EEG data using gamma band analysis and a rapid response handheld device has been demonstrated in a recent study [42]. This study involved 13 critically ill participants and achieved an accuracy greater than 70%, with stepwise discriminant analysis providing the best performance. Regarding delirium detection models, a model developed using single-channel quantitative EEG data and Random Forest classification achieved an AUC of 0.76 in a population of 615 patients, including 286 ICU and 329 non-ICU patients [61]. Additionally, a deep learning approach combining convolutional and recurrent neural networks was used to track consciousness and delirium from frontal EEG signals in 285 ICU patients, achieving an AUC of 0.80 for delirium detection [57]. Overall, integrating ML with EEG data can outperform traditional methods, such as those relying on relative delta power [62], by enabling early identification and intervention.

Previous studies have often been limited by narrower patient populations, fewer EEG variables, or a focus on detection rather than prediction of delirium, reducing the applicability of their findings. The present study evaluated the use of EEG data, independently or combined with demographic, clinical, and laboratory information, for predicting delirium in critically ill patients with SARS-CoV-2 infection, aiming to contribute to the existing research by incorporating specific variables and providing new insights. To further this goal, several EEG-based ML models were developed to explore their potential for improving delirium prediction and management in vulnerable ICU populations.

## Materials and methods

### Study design

This was a prospective observational study conducted in a tertiary ICU setting, analyzing EEG, demographic, clinical, and laboratory data from critically ill patients with SARS-CoV-2 infection to develop ML models for delirium prediction.

### Population

**Fig. 1:**
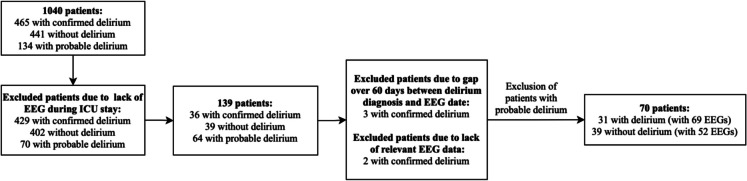
Flowchart of patient selection. *Abbreviations*: EEG, electroencephalography; ICU, Intensive Care Unit

A total of 1040 adult (≥ 18 years) patients admitted to the ICU at Hospital São José in Lisbon, Portugal, were included in this study (Fig. 1).

Out of the 1040 patients, 465 were diagnosed with delirium, 441 did not exhibit delirium, and 134 were suspected of having delirium. Probable delirium was defined as cases in which there was clinical suspicion—based on behavioral signs, medication use (e.g., neuroleptics), or medical chart notes—but where CAM-ICU assessment or formal diagnostic criteria were not consistently applied or documented. Those without an EEG during their ICU stay were excluded, reducing the cohort to 139 patients: 36 with confirmed delirium, 39 without delirium, and 64 with probable delirium. From this group, five patients with delirium were excluded: three because their EEG recordings were conducted more than 60 days prior to the delirium diagnosis, and two due to a lack of relevant EEG information for this study. Furthermore, probable delirium cases were also excluded, resulting in a final cohort of 70 patients: 31 with confirmed delirium (and 69 EEGs) and 39 without delirium (and 52 EEGs).

All patients tested positive for COVID-19 via real-time polymerase chain reaction targeting SARS-CoV-2 and were admitted to the ICU between March 10, 2020, and August 26, 2022. None of the patients were terminally ill or exhibited delirium at ICU admission. Additionally, the patients had no history of major psychiatric disorders or pre-existing neurological conditions that could confound the analysis, and only their first ICU admission during the study period was considered to avoid potential biases from multiple admissions.

Delirium was assessed using the CAM-ICU method [27]. The CAM-ICU score was determined by evaluating the presence of four specific criteria to assess the patient’s cognitive state: acute onset or fluctuating course, inattention, disorganized thinking, and altered level of consciousness. Patients with a Richmond Agitation and Sedation Scale (RASS) [63] score of − 4 or − 5 were considered ineligible for CAM-ICU screening. Those with at least one positive CAM-ICU score during their ICU stay were diagnosed with delirium. In cases in which delirium assessment was not performed, the delirium status was established through a comprehensive review of the clinical notes. This review assessed the use of neuroleptics, the RASS score, and the Diagnostic and Statistical Manual of the American Psychiatric Association (DSM-5) criteria [64].

### Demographic, clinical, and laboratory characteristics

In total, 665 demographic, clinical, and laboratory features were collected. Patient demographics, including sex, age, nationality, and ICU admission characteristics—such as the reason for ICU admission and the Portuguese COVID-19 wave (including six waves of the pandemic) during which the patients were hospitalized—were examined.

The collected clinical variables encompass a broad spectrum of comorbidities, including arterial hypertension, diabetes mellitus, chronic kidney disease, obesity, dyslipidemia, ischemic heart disease, congestive heart failure, chronic respiratory disease, pulmonary hypertension, stroke, solid and hematologic cancers, autoimmune disorders, chronic liver disease, history of organ transplantation, hyperuricemia, and neurological conditions, such as epilepsy, Parkinson’s disease, schizophrenia, multiple sclerosis, and amyloidosis.

Information on key interventions, such as the use of IMV and extracorporeal membrane oxygenation (ECMO), was also collected.

The laboratory parameters acquired were detailed, including blood gas analysis, hemogram, ionogram, coagulation markers (e.g., international normalized ratio (INR), activated partial thromboplastin time), liver function tests (e.g., bilirubin, alkaline phosphatase), kidney function markers (e.g., creatinine, urea), and other biomarkers, such as C-Reactive Protein, ferritin, lactate dehydrogenase, procalcitonin, troponin, and fibrinogen.

Data on medications administered before and during the ICU stay were particularly extensive, covering sedatives (e.g., midazolam, oxazepam), antibiotics, anticoagulants, corticosteroids, immunosuppressants, and other therapeutic classes. COVID-19-specific variables, such as vaccination status and the number of days between symptom onset and ICU admission, were also collected.

Additional variables included ICU length of stay and mortality rates in both the hospital and ICU settings.

### EEG acquisition and analysis

#### EEG acquisition

Routine digital video EEGs were performed at the bedside by qualified EEG technicians using a Brain Quick EEG system with a SD PLUS™ amplifier (Micromed S.p.A., Treviso, Italy). The methodology for EEG recordings adhered to the guidelines of the International Federation of Clinical Neurophysiology for the use of EEG in the ICU [65]. Recordings were obtained using 21 grass gold cup scalp electrodes placed according to the international 10–20 system [66], along with an additional lead for electrocardiogram monitoring.

Before EEG recording, patients were prepared by ensuring their comfort and providing a thorough explanation of the procedure when possible. The EEG equipment was calibrated to ensure accurate recordings, and steps were taken to minimize artifacts, such as securing electrode contacts and reducing electrical interference. The impedances were kept lower than 5 kΩ to ensure high-quality signal acquisition. The EEG recordings lasted for 30 min and included reactivity testing in response to auditory, tactile, and painful stimuli, as well as the alpha reactivity test, which involves asking patients to open and close their eyes, provided they were not profoundly sedated. The recorded EEG data were digitally stored, anonymized, and integrated with the corresponding demographic, clinical, and laboratory data of the same patients.

The data reflecting the patient’s clinical condition at the time of EEG recording included medication type and dosage, type of mechanical ventilation, Glasgow Coma Scale (GCS), and RASS score.

#### EEG timeline

EEG recordings were assessed at specific time intervals to monitor changes and treatment responses, providing comprehensive insights into the patient’s neurological status. The EEGs were conducted at the following time points:

- Initial assessment at ICU admission: a baseline EEG was performed to evaluate the initial neurological status, identify any pre-existing abnormalities, and establish a reference for subsequent comparisons.
- Post-sedation: an EEG was conducted after sedation to assess the effects of sedative agents on brain activity and distinguish sedation-induced changes from underlying pathological patterns.
- Follow-up: periodic EEGs were performed based on clinical evolution and patient-specific needs, to track the progression of EEG patterns, detect emerging complications (e.g., seizures or encephalopathy), and assess treatment efficacy. The frequency of these follow-ups was guided by the patient’s neurological status, with closer monitoring in cases of deterioration or abnormal findings.
- Final assessment: a final EEG was performed to evaluate the overall neurological outcome and recovery trajectory, aiding in prognosis and long-term management decisions.

During the initial assessment, none of the patients were diagnosed with delirium, although it could develop in subsequent evaluations. Due to the limited time patients spent in the ICU, it was not always possible to perform every EEG at each interval; however, each patient underwent at least one EEG at ICU admission.

This study primarily aimed to predict delirium early at ICU admission, with most models developed using EEG data collected during the initial assessment. Temporal analyses were only incorporated in the final model to evaluate EEG changes before and after the delirium onset.

#### EEG analysis

Each EEG was independently analyzed by two senior neurophysiologists in a single-blind manner, as they were blinded to the patient’s clinical data. EEG interpretation was performed according to the 2021 American Clinical Neurophysiology Society (ACNS) nomenclature [67] by assessing the following parameters:

- Background EEG:
  o Symmetry: classified as symmetric, mildly asymmetric (consistent asymmetry in amplitude < 50% or frequency difference of 0.5–1 Hertz (Hz)), or markedly asymmetric (amplitude > 50% difference or frequency difference > 1 Hz).
  o Predominant background frequency: defined as delta (< 4 Hz), theta (4–7 Hz), alpha (8–13 Hz), and/or beta (> 13 Hz). If multiple frequency bands were equally prominent, each was recorded.
  o Posterior Dominant Rhythm (PDR): categorized as present, absent, or unclear, with its exact frequency recorded to the nearest 0.5 Hz when present.
  o Continuity: described as continuous, nearly continuous, discontinuous, burst-suppression, burst-attenuation, or suppression, based on the proportion of the EEG record affected. Continuous activity had no interruptions, while nearly continuous activity contained occasional suppression or attenuation affecting 1–9% of the record. Discontinuous activity involved 10–49% suppression or attenuation. Burst-suppression was defined as 50–99% suppression (< 10 microvolts (µV)), alternating with bursts of activity lasting ≥ 0.5 s and containing at least four phases. Burst-attenuation followed the same criteria as burst-suppression but occurred in the attenuation range (≥ 10 µV but < 50% of background activity). Finally, suppression was characterized by > 99% of the EEG record showing suppression (< 10 µV).
  o Reactivity: categorized as reactive, unreactive, unclear, unknown, or stimulus-induced rhythmic, periodic, or ictal-appearing discharges (SIRPIDs-only).
  o State changes: classified as present with normal N2 transients, present with abnormal transients, present but without N2 transients, or absent. State changes were defined as sustained EEG background variations related to alertness or stimulation, persisting for at least 60 s.
  o Cyclic Alternating Pattern of Encephalopathy (CAPE): classified as present, absent, or unknown. CAPE was defined as spontaneous, alternating background patterns lasting at least 10 s each, repeating regularly for at least 6 cycles.
  o Voltage: classified into four categories, namely, high (≥ 150 µV), normal (> 20 to < 150 µV), low (10 to < 20 µV) and suppressed (< 10 µV).
  o Anterior-posterior (AP) gradient: classified as present, absent, or reversed. Defined as a persistent gradient where lower amplitude, faster frequencies occur anteriorly, and higher amplitude, slower frequencies occur posteriorly.
  o Breach effect: determined as present or absent. If present, the location was recorded.
- Sporadic Epileptiform Discharges (SEDs): defined as nonrhythmic and nonperiodic spikes, polyspikes, and sharp waves. These were quantified based on their occurrence rate and categorized as abundant (≥ 1 per 10 s, but not periodic), frequent (≥ 1 per minute but < 1 per 10 s), occasional (≥ 1 per hour but < 1 per minute), or rare (< 1 per hour).
- Rhythmic or Periodic Patterns (RPPs): PDs, rhythmic delta activity (RDA), or spike-wave patterns were classified as generalized, lateralized, bilateral independent, unilateral independent, or multifocal. The prevalence, duration, frequency, number of phases, sharpness, and voltage were assessed, with any stimulus-induced, stimulus-terminated, evolving, or fluctuating patterns noted.
- Electrographic seizures (ESz), Electroclinical Seizures (ECSz), Electrographic Status Epilepticus (ESE), and Electroclinical Status Epilepticus (ECSE): ESz, ECSZ, ESE, and ECSE were defined based on the discharge rate, pattern evolution, and duration. The classification considered both clinical manifestations and EEG response to treatment. However, for convenience and simplification of the analysis—since no cases of ESz or ECSz were recorded—these categories were grouped under the single designation Status Epilepticus (SE).
- Brief Potentially Ictal Rhythmic Discharges (BIRDs): BIRDs were defined as rhythmic activity > 4 Hz lasting ≥ 0.5 to < 10 s, with focal, lateralized, bilateral independent, unilateral independent, or generalized distribution. BIRDs were identified when they exhibited evolution, similarity to interictal discharges, or sharply contoured waveforms.
- Ictal-Interictal Continuum (IIC): patterns that did not meet the criteria for definite seizure but had ictal-like features were categorized within the IIC. This included periodic discharges (PDs) > 1 Hz, rhythmic delta activity (RDA), or spike-wave patterns that had a “plus” modifier (+ F, + S, or + R) or fluctuated over time. These patterns were considered to potentially contribute to impaired alertness, clinical symptoms, or neuronal injury.

In total, 32 EEG features were collected (Table S1).

Throughout the remainder of this paper, the terms “features” and “variables” may be used interchangeably.

This study focused on analyzing the EEG features extracted from clinical reports rather than raw signals. This approach was deliberately chosen to align with the established procedures of the ICU where the data was collected, guaranteeing that normal workflows were neither disrupted nor altered. By prioritizing clinically validated metrics, this method maintained the practical relevance and immediate interpretability of the extracted features within a clinical context. This methodology is consistent with prior research and builds upon existing findings, facilitating predictive modeling and ensuring practical applicability in real-world settings.

### Statistical analysis

For continuous variables, the independent samples t-test was used to compare the two groups when the data followed a normal distribution. In instances in which the data were non-normally distributed, the Mann–Whitney *U* test served as a non-parametric alternative. For categorical data analysis, chi-square tests were used to evaluate independence within the contingency tables. If the contingency table included cells with low expected frequencies, potentially compromising the validity of the chi-square results, Fisher’s exact test was applied. Statistical significance was established using a two-sided *p-*value of less than 0.05.

Descriptive and inferential statistics were performed using IBM SPSS Statistics software, version 27 (IBM Corp., New York, NY, USA).

### ML and data analysis

ML algorithms and feature selection were conducted using Orange 3, version 3.19.0 (Bioinformatics Lab, University of Ljubljana, Slovenia) [68].

Several ML algorithms were evaluated, including Naïve Bayes, Logistic Regression, a Decision Tree, a Support Vector Machine (SVM), and Random Forest. Among these, the model with the best performance was selected for detailed analysis, demonstrating superior results across key metrics, such as area under the curve (AUC), accuracy, precision, sensitivity, and specificity. These metrics were selected to provide a comprehensive assessment of the model’s ability to classify delirium and non-delirium cases. Given the potential class imbalance, AUC was considered as a primary metric due to its ability to assess the model’s discrimination across all decision thresholds. However, sensitivity and specificity were also evaluated to ensure balanced performance and clinical applicability.

Each of the evaluated models offers distinct characteristics and advantages. Naïve Bayes, a probabilistic classifier, uses Bayes’ theorem to estimate class probabilities, operating under the assumption of feature independence [69–71]. Logistic Regression is a popular linear model that predicts binary outcomes using a logistic function and is valued for its interpretability and efficiency [72]. Decision Trees, which are rule-based models that partition data into hierarchical branches, are intuitive and well-suited for capturing nonlinear relationships [73]. Support Vector Machines (SVMs) identify the hyperplane that maximizes the margin between classes and are particularly effective for high-dimensional or complex data when paired with appropriate kernels [74]. Finally, Random Forests, an ensemble method that combines multiple decision trees, improves generalizability and robustness by reducing overfitting by averaging predictions across trees [75, 76].

From the initial variables collected, the Information Gain feature scoring method was applied to identify the most relevant features for discriminating the target variable (delirium) from the control group (non-delirium). The Information Gain was selected for feature selection due to its ability to evaluate the relevance of individual features in distinguishing between target classes [77, 78]. To ensure unbiased model evaluation and to prevent data leakage, feature selection was incorporated into the cross-validation process. Using this method, the features that most significantly contributed to model performance were identified and used to address all the research questions. Performance metrics were obtained using tenfold stratified cross-validation, where the data were divided into 10 subsets. In each iteration, one subset (10%) was used for testing, while the remaining nine subsets (90%) were used for training.

Confusion matrices were used to evaluate the performance of the classification model, providing detailed information about the true positives, false positives, true negatives, and false negatives [79]. True positives and true negatives refer to correctly identified cases of delirium and non-delirium, respectively. Sensitivity measures the proportion of true positive cases (delirium) correctly identified, whereas specificity measures the proportion of true negative cases (non-delirium) correctly identified. These techniques provide valuable insights into the data structure and clustering, which are essential for interpreting EEG features in delirium prediction.

To enhance the interpretability and transparency of the models, several nomograms were developed for both Naïve Bayes and Logistic Regression. These nomograms visually represent the impact of individual features on the predicted probability of a binary outcome (0 or 1) [80]. For binary features, it shows how the presence (1) or absence (0) of a feature can tip the prediction toward one side of the target variable. For continuous features, the nomogram creates bins representing different value ranges that reflect the predictive influence of each bin. This binning process allows a detailed understanding of how varying levels of continuous features affect the model’s predictions [80, 81]. Users can interact with the nomogram to observe how changes in the feature values influence the predicted outcome, which enhances the clarity and comprehensibility of the model.

#### Feature selection

To ensure the quality and relevance of the data used in the ML models, a set of rigorous criteria was applied in selecting both categorical and continuous variables. This process aimed to eliminate features with low variability, insufficient representativeness, or a high proportion of missing values, as described below:

- Low variability in the categorical variables:
  o Criteria: categorical variables in which a single category (0 or 1) dominated more than 95% of the cases in both groups were excluded.
  o Objective: to ensure that the variables exhibit sufficient variability to distinguish between the analyzed groups (delirium vs. non-delirium).
- Low frequency and absence in categorical variables:
  o Criteria: the variables for which one category (0 or 1) had fewer than two absolute occurrences in either group were removed; the variables for which one category was entirely absent in one group were also excluded.
  o Objective: to ensure that all categorical variables have minimal representativeness in both groups to avoid statistical bias.
- High frequency of missing values in the continuous variables:
  o Criterion: continuous variables with more than 30% missing values were excluded.
  o Objective: to retain only variables with at least 70% valid values and to minimize the impact of missing data on modeling.
- Low variance in the continuous variables:
  o Criterion: continuous variables with a variance of less than 0.01 were removed.
  o Objective: to eliminate features with low dispersion, as they have limited predictive value for classification between the groups.

These criteria were implemented as part of the data selection pipeline using the Orange 3 software, ensuring that only the most relevant and representative features were included in the subsequent modeling stages. By filtering out low-quality features and improving data efficiency, this process enhanced the predictive performance of the models while retaining critical information for analysis.

## Results

### General characteristics of the population at ICU admission

Out of the 70 patients considered, 31 (44.3%) were diagnosed with delirium (Fig. 1). At ICU admission, delirium development was significantly associated with male sex (*p* = 0.011) and non-administration of the COVID-19 vaccine (*p* = 0.009) (Table 1).

**Table 1:** Demographic and clinical characteristics of 70 patients, including 31 patients diagnosed with delirium, and the *p*-value of the statistical analysis comparing the two groups. *Abbreviations:* ECMO, extracorporeal membrane oxygenation; ICU, Intensive Care Unit; IMV, invasive mechanical ventilation; IQR, interquartile range

	Patients with confirmed delirium (*n* = 31)	Patients without delirium (*n* = 39)	*p*-value	Statistic test
Age (years) (median, IQR)	51.00 (33.00)	58.00 (30.00)	0.615	Mann–Whitney *U*
Male sex (n, %)	28 (90.3%)	25 (64.1%)	0.011	Chi-square
Portuguese nationality (n, %)	19 (61.3%)	27 (69.2%)	0.659	Chi-square
Administration of the COVID-19 vaccine (n, %)	7 (22.6%)	22 (56.4%)	0.009	Chi-square
Comorbidities (n, %)	24 (77.4%)	31 (79.5%)	1.000	Chi-square
Arterial Hypertension (n, %)	12 (38.7%)	18 (46.2%)	0.702	Chi-square
Diabetes Mellitus (n, %)	6 (19.4%)	7 (17.9%)	1.000	Chi-square
Dyslipidemia (n, %)	7 (22.6%)	10 (25.6%)	0.987	Chi-square
Obesity (n, %)	10 (32.3%)	13 (33.3%)	1.000	Chi-square
Hospital death (n, %)	4 (12.9%)	9 (23.1%)	0.437	Chi-square
ICU death (n, %)	1 (3.2%)	7 (17.9%)	0.122	Chi-square
Days of ICU stay (median, IQR)	16.00 (13.00)	6.00 (3.00)	< 0.001	Mann–Whitney *U*
Use of IMV (n, %)	23 (74.2%)	21 (53.8%)	0.133	Chi-square
Use of ECMO (n, %)	4 (12.9%)	4 (10.3%)	1.000	Chi-square
Propofol (n, %)	21 (67.7%)	18 (46.2%)	0.118	Chi-square
Midazolam (n, %)	11 (35.5%)	6 (15.4%)	0.095	Chi-square

Delirium patients also had a significantly longer ICU stay (median of 16.00 days) compared with non-delirium patients (median of 6.00 days, *p* < 0.001). These findings are consistent with those of the existing literature indicating that factors such as sex, COVID-19 vaccination, and length of ICU stay are critical determinants of the development and severity of delirium [82–84]. Other factors, such as age, comorbidities, ICU and hospital death, obesity, arterial hypertension, diabetes mellitus, dyslipidemia, use of ECMO or IMV, or medications (e.g., propofol, and midazolam), did not significantly differ between the groups.

Most patients (*n* = 63, i.e., 90%) were admitted to the ICU primarily because of acute respiratory failure caused by SARS-CoV-2 infection (Table S2). Furthermore, analysis of the infection waves in Portugal revealed no statistically significant differences in the distribution of infection waves between delirium and non-delirium patients (Table S3). Nevertheless, while most patients in this study were admitted during the fourth wave, accounting for 57.1% of all cases, a study on the same initial population of 1,040 patients found that the majority were actually admitted during the second and third waves of the pandemic [85], similar to trends observed in other countries.

### EEG-based ML models

Various ML models were developed to predict delirium, each employing a distinct approach.

Initially, traditional and well-known EEG variables associated with delirium development were analyzed both independently (individual EEG models) and collectively (combined EEG model). Subsequently, all collected EEG variables were considered and evaluated using a ranking score method (comprehensive EEG model). Demographic, clinical, and laboratory variables were then incorporated to assess potential improvements in model performance (model based on EEG, demographic, clinical and laboratory variables). For all these approaches, EEG data recorded at ICU admission were used, involving 70 selected patients (31 with confirmed delirium and 39 without delirium).

Lastly, a final model was developed to identify differences in EEG features before (at ICU admission) and after the diagnosis of delirium (model based on temporal EEG changes), using 22 EEGs from 11 male patients with confirmed delirium, each having EEGs recorded both before and after the delirium diagnosis.

For clarity, these models are referred to by their respective designations, as provided above, throughout the remainder of this paper.

#### Individual and combined delirium prediction models using literature-supported EEG variables

A preliminary exploration of EEG recordings aimed to differentiate between critically ill patients with and without delirium based on EEGs performed at ICU admission. The analysis included the selected cohort of 70 patients, 31 with confirmed delirium and 39 without.

Initially, 12 EEG features known to be associated with delirium were selected based on an extensive review of the literature and key studies. These features included increased delta activity (predominant background frequency of less than 4 Hz) [40, 51, 82, 83, 86], increased theta activity (predominant background frequency of 4 to 7 Hz) [47, 82, 83, 86], reduction in alpha activity [40, 47, 82, 83], absent reactivity [51, 87], RPPs [41, 51, 86, 88], low voltage (< 20 µV) [89], suppression [56, 90], burst-suppression/burst-attenuation [41, 47], absent PDR [86, 91], SEDs [40, 41, 47], triphasic waves [83, 86], and PDA [50, 83]. Given that none of the patients in the present study had triphasic waves or suppression, these features were excluded from the analysis, thereby reducing the set to 10 EEG features.

Various ML models were employed to assess the individual predictive power of the 10 EEG features for delirium, with the results of the best-performing model for each variable presented in Table 2.

**Table 2:** Performance metrics from the tenfold cross-validation of individual EEG feature-based models for delirium prediction in critically ill patients. EEG variables were selected and ranked according to their predictive value using the Information Gain scoring method. The reported metrics include AUC, accuracy, precision, sensitivity, and specificity for each EEG variable analyzed. *Abbreviations:* AUC, area under the curve; EEG, electroencephalography; PDR, posterior dominant rhythm; PDA, polymorphic delta activity; RPPs, rhythmic or periodic patterns; SEDs, sporadic epileptiform discharges; SVM, support vector machine

Variable	Model	AUC	Accuracy	Precision	Sensitivity	Specificity
Absence of reactivity	Random Forest	0.488	0.543	0.000	0.000	0.974
Reduction in alpha activity	SVM	0.483	0.557	0.000	0.000	1.000
Burst-suppression/burst-attenuation	SVM	0.463	0.557	0.000	0.000	1.000
PDA	Naïve Bayes	0.529	0.486	0.143	0.032	0.846
SEDs	Logistic Regression	0.525	0.557	0.000	0.000	1.000
Absence of PDR	Naïve Bayes	0.592	0.514	0.440	0.355	0.641
Low voltage (< 20 μV)	Naïve Bayes	0.533	0.586	0.667	0.129	0.949
Predominant background frequency of less than 4 Hz	Naïve Bayes	0.600	0.614	0.591	0.419	0.769
RPPs	Naïve Bayes	0.558	0.600	0.667	0.194	0.923
Predominant background frequency of 4 to 7 Hz	Naïve Bayes	0.667	0.643	0.565	0.839	0.487

The model using a predominant background frequency of 4 to 7 Hz (increased theta activity) as a predictor achieved the highest AUC (0.667) among the individual EEG feature models. However, it is unbalanced by standard performance metric definitions, strongly favoring sensitivity (0.839)—indicating it captured most actual delirium cases—over specificity (0.487), which increased the rate of false positives. While this trade-off represents an improvement compared to the other models in this study, it highlights a limitation that must be carefully managed in clinical applications. Additionally, the model correctly classified 64.3% of cases, achieving an accuracy of 0.643, and had a precision of 0.565, meaning that 56.5% of its positive predictions were correct.

In comparison, the second-best model, corresponding to a predominant background frequency of less than 4 Hz, had a lower AUC (0.600), accuracy (0.614), and sensitivity (0.419) but a higher specificity (0.769), making it more suitable for minimizing false positives. Although imbalanced, according to the standard performance metric, this variation may be acceptable in applications prioritizing the reduction of false positives.

The other individual models demonstrated notably lower performance metrics. For instance, the model using absent reactivity as a predictor feature had an AUC of 0.488, accuracy of 0.543, precision of 0.000, sensitivity of 0.000, and specificity of 0.974. Despite its high specificity, these poor metrics—particularly the near-random AUC and complete inability to identify true positive cases—reflect the limited predictive power of this individual feature.

Additionally, a considerable number of individual EEG models (i.e., absent reactivity, reduction in alpha activity, burst-suppression/burst-attenuation, PDA, SEDs, low voltage, and RPPs) displayed high specificity, correctly identifying most negative cases, but their sensitivity was low, failing to detect more than half of the true positive cases. Even models with moderate AUCs, such as low voltage or RPPs, had sensitivity below 0.5, limiting their reliability in identifying delirium. This pattern highlights the inherent challenges of using single EEG variables to capture the complexity of this condition and underscores their limited practicality for clinical use, particularly in ICU settings where identifying delirium cases is critical.

Overall, the analysis highlights the difficulty in optimizing both sensitivity and specificity for most EEG features. Although several variables demonstrate high specificity, effectively avoiding false positives, they often lack sensitivity, failing to detect many true delirium cases. In addition, most models’ AUCs fall within the poor-to-fair range, further emphasizing the limited predictive power of individual features. A multivariable approach or integration of these features into a more complex predictive model could improve diagnostic accuracy and reliability, reducing both false positives and false negatives.

To further assess the predictive performance of the selected literature-supported EEG variables for delirium, a combined model was developed by incorporating all 10 identified EEG features. The features were then ranked, and only those that significantly enhanced the model’s accuracy were retained (Table 3).

**Table 3:** Performance metrics from the tenfold cross-validation of the Logistic Regression combined EEG model for delirium prediction in critically ill patients. EEG variables were ranked based on their predictive contribution using the Information Gain scoring method. The reported metrics include AUC, accuracy, precision, sensitivity, and specificity. *Abbreviations:* AUC, area under the curve; EEG, electroencephalography; PDR, posterior dominant rhythm; RPPs, rhythmic or periodic patterns

RANK (order)	Model	AUC	Accuracy	Precision	Sensitivity	Specificity
1. Predominant background frequency of 4 to 7 Hz	Logistic Regression	0.700	0.643	0.615	0.516	0.744
2. RPPs						
3. Predominant background frequency of less than 4 Hz						
4. Low voltage (< 20 μV)						
5. Absence of PDR						

The combined model, developed using Logistic Regression, achieved an AUC of 0.700. Although slightly higher than the best individual AUC (0.667, achieved by the model using predominant background frequency of 4 to 7 Hz), this difference does not represent a substantial improvement.

This inclusive approach prioritized five EEG features: (1) predominant background frequency of 4 to 7 Hz, (2) RPPs, (3) predominant background frequency of less than 4 Hz, (4) low voltage, and (5) PDR, specifically its absence (Figure S1). By incorporating these features, the combined model demonstrated an accuracy of 0.643, matching the best-performing individual model and outperforming others, such as low voltage (0.586) and predominant background frequency of less than 4 Hz (0.614). It achieved a precision of 0.615, specificity of 0.744, and sensitivity of 0.516. While its sensitivity was lower than that of the best-performing individual model (0.839), it exceeded most others and offered a modest improvement in specificity compared with the best individual model (0.487), although it remained below features with exceptionally high specificity, such as absent reactivity (0.974) and low voltage (0.949). Overall, the combined model demonstrated a better balance between sensitivity and specificity than the individual models, which can be useful in settings where false positives can be tolerated to reduce false negatives.

The confusion matrix (Table S4) provides further insight into the model’s predictive performance and highlights its limitations. Among the 39 negative cases, 29 were true negatives and 10 were false positives, contributing to a specificity of 0.744. Similarly, out of the 31 positive cases, 16 were true positives and 15 were false negatives, resulting in a sensitivity of 0.516. Although the model reduced the overprediction of delirium compared with the individual models, it failed to identify a significant proportion of true delirium cases, which could result in underdiagnosis in ICU scenarios where early detection of delirium is vital for patient outcomes.

In conclusion, although the combined EEG features model demonstrated a modestly better overall performance compared with the individual models, the combination of EEG-specific variables alone still provided limited diagnostic accuracy.

#### Delirium prediction model using comprehensive EEG variables

Subsequently, an advanced model leveraging all 32 collected EEG variables (Table S1) was developed to identify differences in EEG recordings between critically ill COVID-19 patients with and without delirium. The same two groups of patients, with EEGs recorded at ICU admission, were used as in previous models.

After the feature selection process, categorical variables with low variability, low frequency, or absence in one group, as well as continuous variables with > 30% missing values or low variance, were removed, leaving 22 of the original set of 32 features for model development.

Five key EEG features were identified based on their predictive contribution, as determined by the Information Gain scoring method, namely: a predominant background frequency of 4 to 7 Hz (increased theta activity), absence of state changes, absence of normal voltage, absence of a predominant background frequency of 8 to 13 Hz, and presence of RPPs (Table 4).

**Table 4:** Performance metrics from the tenfold cross-validation of the Naïve Bayes comprehensive EEG model for delirium prediction in critically ill patients. EEG variables were ranked based on their predictive contribution using the Information Gain scoring method. The reported metrics include AUC, accuracy, precision, sensitivity, and specificity. *Abbreviations:* AUC, area under the curve; EEG, electroencephalography; RPPs, rhythmic or periodic patterns

RANK (order)	Model	AUC	Accuracy	Precision	Sensitivity	Specificity
1. Predominant background frequency of 4 to 7 Hz	Naïve Bayes	0.733	0.671	0.625	0.645	0.692
2. Absence of EEG state changes						
3. Low voltage (< 20 μV)						
4. Absence of a predominant background frequency of 8 to 13 Hz						
5. RPPs						

By integrating all the collected EEG features, the model achieved an AUC of 0.733, which was the highest value obtained among all models relying exclusively on EEG data. The model also reached an accuracy of 0.671, correctly classifying 67.1% of the cases, which is only slightly better than models with fewer EEG features, such as the combined Logistic Regression model (accuracy of 0.643). Although this represents an improvement over previous models, the difference is modest and highlights the challenges of achieving significant predictive gains by merely expanding the set of EEG features.

From the confusion matrix (Table S5), 27 of the 39 actual negative cases were correctly classified as negatives (true negatives), while 12 were misclassified as positives (false positives). Among the 31 actual positive cases, the model correctly identified 20 true positives but missed 11 false negatives. Compared with the combined Logistic Regression model (with sensitivity of 0.516, and specificity of 0.744), this model demonstrated better sensitivity at the cost of reduced specificity. This trade-off reflects an improved ability to detect true delirium cases, but the increased false positives may pose challenges in clinical settings.

While the model showed improved predictive performance, particularly in sensitivity, the modest gains in AUC and accuracy highlight the limitations of relying solely on EEG features for delirium prediction. These results suggest diminishing returns from adding more features to the model, emphasizing the need for further refinement, such as integrating EEG data with clinical variables, to achieve more meaningful improvements in diagnostic accuracy and reliability.

**Fig. 2:**
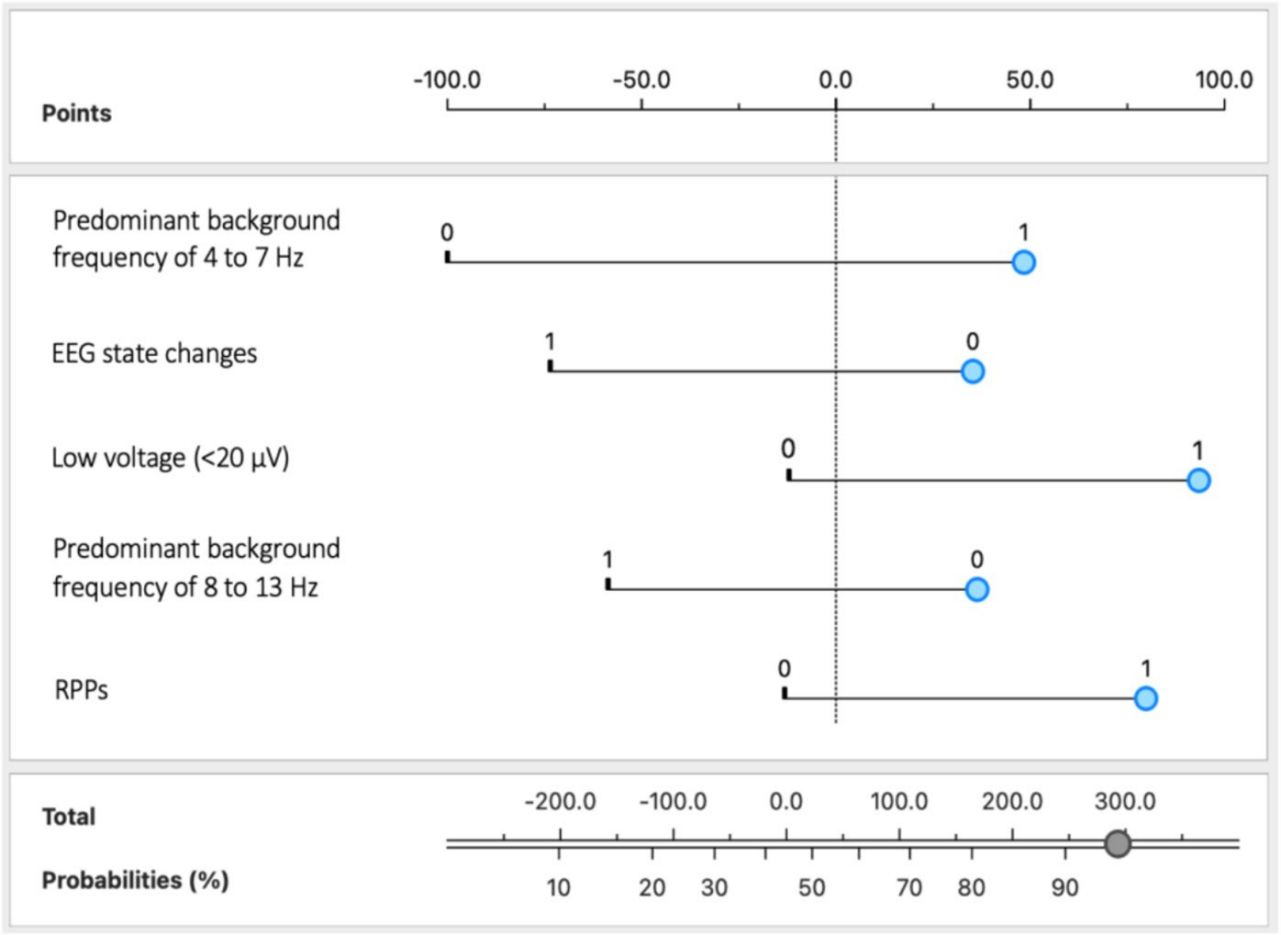
Nomogram of the delirium prediction model using comprehensive EEG variables. The variables were categorized as follows: predominant background frequency of 4 to 7 Hz, EEG state changes, low voltage (**< **20 µV), predominant background frequency of 8 to 13 Hz, and RPPs were indicated by 1 for present and 0 for absent. *Abbreviations:* EEG, electroencephalography; RPPs, rhythmic or periodic patterns

A nomogram was developed to visually represent the importance of the five key EEG findings in predicting delirium in critically ill patients (Fig. 2).

To use the nomogram, each feature is given a value based on its presence or level, and the blue markers indicate the values or categories associated with delirium development in this study cohort. For instance, a patient with an EEG with a predominant background frequency of 4 to 7 Hz (1), absence of state changes (0), absence of normal voltage (0), absence of a predominant background frequency of 8 to 13 Hz (0), and presence of RPPs (1) would have a predicted probability of approximately 93% for developing delirium. Ultimately, by integrating multiple EEG features into a single visual tool, this nomogram offers an accessible and practical method for clinicians to identify patients at a higher risk of developing delirium.

#### Delirium prediction model based on EEG, demographic, clinical and laboratory variables

To explore the impact of including additional variables, demographic, clinical, and laboratory factors (665 features) were combined with the 32 initially collected EEG variables. This approach aimed to determine whether these additions would influence the model’s performance.

After data refinement, the feature set was reduced to 127 variables (22 EEG features and 105 demographic, clinical or laboratory features—Table S6) by removing those with low variability, frequency, or high missing data.

As in the previously developed models, two patient groups were used, each consisting of 31 delirium patients and 39 non-delirium patients with EEGs recorded at ICU admission.

From the refined feature set, the algorithm identified five key factors associated with delirium development at ICU admission: predominant background frequency of 4 to 7 Hz (increased theta activity), male sex, non-administration of COVID-19 vaccination, administration of sodium chloride, and absence of EEG state changes (Table 5).

**Table 5:** Performance metrics from the tenfold cross-validation of the Logistic Regression model incorporating EEG, demographic, clinical, and laboratory variables for delirium prediction in critically ill patients. EEG, demographic, clinical, and laboratory variables were ranked based on their predictive contribution using the Information Gain scoring method. The reported metrics include AUC, accuracy, precision, sensitivity, and specificity. *Abbreviations:* AUC, area under the curve; COVID-19, Coronavirus Disease 2019; EEG, electroencephalography

RANK (order)	Model	AUC	Accuracy	Precision	Sensitivity	Specificity
1. Predominant background frequency of 4 to 7 Hz	Logistic Regression	0.825	0.714	0.704	0.613	0.795
2. Male sex						
3. Non-administration of the COVID-19 vaccination						
4. Administration of sodium chloride						
5. Absence of EEG state changes						

The updated model achieved an AUC of 0.835, which is the highest among all tested models. This represents an improvement over the best individual EEG model (AUC = 0.667, Table 2), the combined Logistic Regression EEG model (AUC = 0.700, Table 3), and the comprehensive Naïve Bayes EEG model (AUC = 0.733, Table 4). The accuracy of 0.714 also surpasses the previous combined and comprehensive models (Tables 3 and 4: 0.643 and 0.671, respectively), demonstrating the value of demographic, clinical, and laboratory variables.

The model exhibited a specificity of 0.795, reflecting a stronger ability to avoid false positives while maintaining a sensitivity of 0.613, which was competitive with the comprehensive EEG model (sensitivity = 0.645, Table 4). This balance between sensitivity and specificity makes the updated model a more practical tool for ICU settings, where false positives may be less concerning than missing true delirium cases.

According to the confusion matrix (Table S7), the model correctly classified 31 out of 39 non-delirium cases (true negatives) and 19 out of 31 delirium cases (true positives), resulting in 8 false positives and 12 false negatives. Although the slight decrease in sensitivity compared with the best individual EEG model may increase the risk of missed delirium cases, the improvement in specificity suggests fewer unnecessary interventions.

By integrating EEG data with demographic, clinical, and laboratory variables, this model offers a more practical and enhanced approach to delirium prediction. The inclusion of factors such as sex, COVID-19 vaccination status, and sodium chloride administration expands the model’s predictive scope beyond EEG features alone, while also addressing potential confounding factors to prevent bias from underlying patient characteristics. This approach highlights the importance of combining multimodal data sources to enhance predictive accuracy and clinical applicability.

**Fig. 3:**
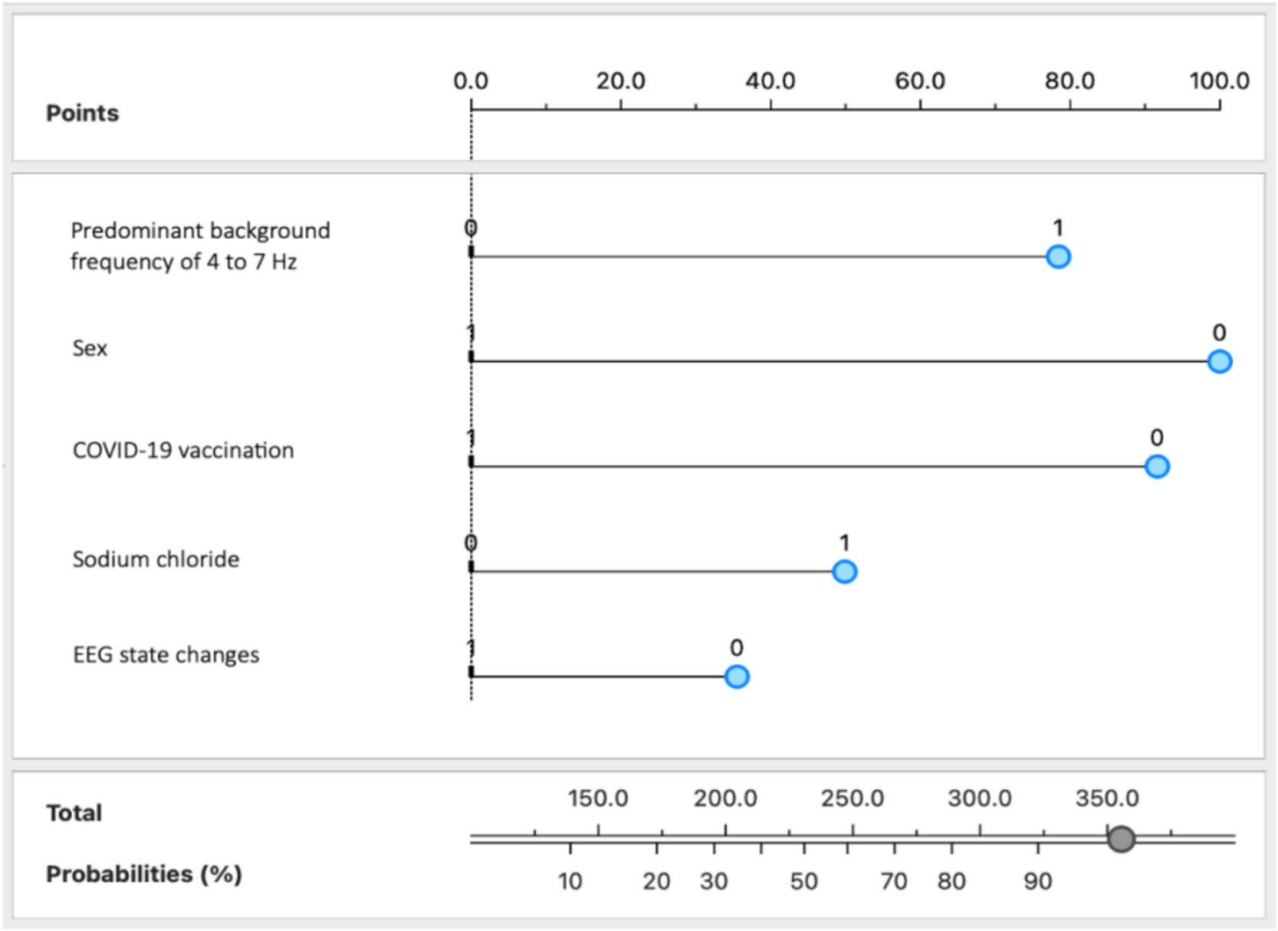
Nomogram for the delirium prediction model using EEG, demographic, clinical, and laboratory variables. The variables were categorized as follows: predominant background frequency of 4 to 7 Hz, COVID-19 vaccination, administration of sodium chloride, and EEG state changes were indicated by 1 for present and 0 for absent; sex was categorized as 1 for female and 0 for male. *Abbreviations:* COVID-19, Coronavirus Disease 2019; EEG, electroencephalography

A nomogram was developed to visually represent the importance of the five key EEG and demographic, clinical, or laboratory features in predicting delirium in critically ill patients (Fig. 3).

To use the nomogram, each feature is given a value based on its presence or level, and the blue markers indicate the values or categories associated with delirium development in this study cohort. For instance, a patient with an EEG with a predominant background frequency of 4 to 7 Hz (1), male sex (0), no COVID-19 vaccine administration (0), administration of sodium chloride (1), and absence of state changes in EEG (0) would have a predicted probability of approximately 95% for developing delirium.

#### Delirium prediction model based on temporal EEG changes

A final model was developed to identify differences in the EEG features before and after delirium diagnosis. This study involved 22 EEGs from 11 male patients with delirium, each having EEGs recorded before (at ICU admission) and after delirium diagnosis. The interval between the first and last EEG ranged from 4 to 54 days, with an average of 15.82 ± 13.05 days. The median patient age was 46.82 ± 15.55 years.

After the feature selection process, categorical variables with low variability, infrequent occurrences, or absence in one group, as well as continuous variables with 30% missing values or low variance, were excluded. This reduced the initial set of 32 EEG features to 7 for model development.

Among all the ML models tested, a Naïve Bayes model prioritizing five key EEG variables, namely PDR, predominant background frequency of 8 to 13 Hz, AP gradient, continuous EEG, and predominant background frequency of 4 to 7 Hz, demonstrated the best performance. The model achieved an AUC of 0.950 and an accuracy, sensitivity, precision, and specificity of 0.818, indicating highly accurate discrimination between pre- and post-diagnosis states (Table 6).

**Table 6:** Performance metrics from the tenfold cross-validation of the Naïve Bayes model for delirium prediction using temporal EEG changes in critically ill patients. EEG features were ranked based on their predictive value using the Information Gain scoring method. The reported metrics include AUC, accuracy, precision, sensitivity, and specificity. *Abbreviations:* AP, anterior–posterior; AUC, area under the curve; EEG, electroencephalography; PDR, posterior dominant rhythm

**RANK (order)**		AUC	Accuracy	Precision	Sensitivity	Specificity
1. Absence of PDR	Naïve Bayes	0.950	0.818	0.818	0.818	0.818
2. Predominant background frequency of 8 to 13 Hz						
3. AP gradient						
4. Continuous EEG						
5. Predominant background frequency of 4 to 7 Hz						

The confusion matrix (Table S8) revealed that of the 11 EEGs recorded after the delirium diagnosis, 9 were correctly classified as “after” (true positives), while two were misclassified as “before” (false negative). Similarly, among the 11 EEGs recorded before the diagnosis, 9 were correctly classified as “before” (true negatives), while two were misclassified as “after” (false positives).

**Fig. 4:**
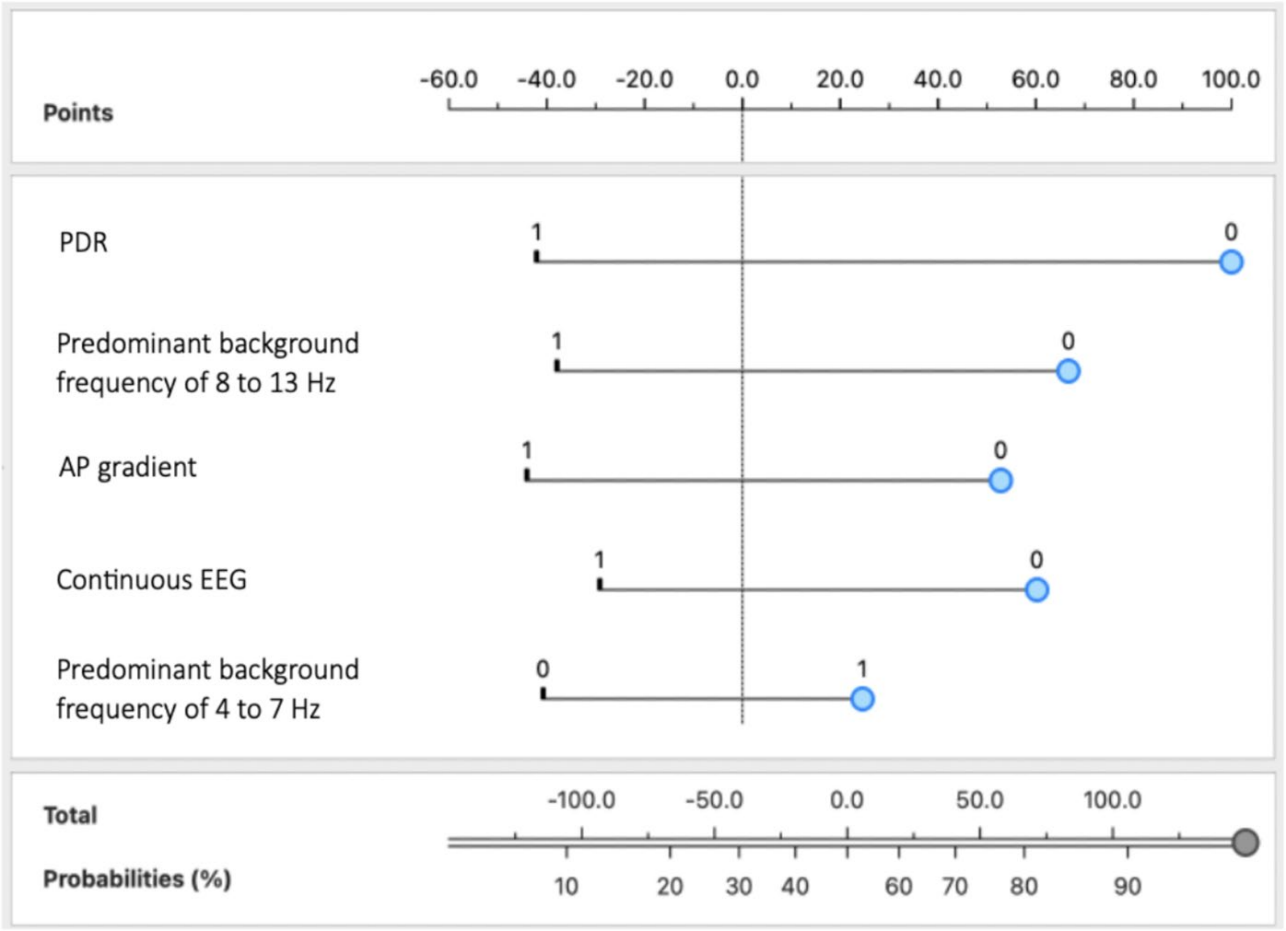
Nomogram for the delirium prediction model based on temporal EEG changes targeting EEG changes before delirium diagnosis. The variables were categorized as follows: PDR, predominant background frequency of 8 to 13 Hz; AP gradient, continuous EEG; and predominant background frequency of 4 to 7 Hz were indicated by 1 for present and 0 for absent. *Abbreviations:* AP, anterior–posterior; EEG, electroencephalography; PDR, posterior dominant rhythm

The interval between the delirium diagnosis and the post-EEG evaluation ranged from 1 to 29 days, with an average of 7.45 ± 7.24 days. EEGs conducted before the diagnosis of delirium revealed that its development was associated with the absence of PDR, predominant background frequency of 8 to 13 Hz, AP gradient, and continuous EEG activity. Additionally, it was linked to the presence of a predominant background frequency of 4 to 7 Hz (Fig. 4). EEG analysis after delirium diagnosis revealed significant shifts in brain activity, including the re-emergence of PDR, acceleration of predominant background frequencies, restoration of the AP gradient, and reappearance of continuous EEG activity (Figure S2). These findings suggest partial restoration of cortical activity as delirium begins to resolve or is treated, supporting previous knowledge that EEG patterns typically normalize as the condition subsides [40, 83, 92–94].

This model highlights the value of the temporal EEG analysis in monitoring delirium progression and recovery. Compared with earlier models relying on static EEG, demographic, clinical, and laboratory variables, the incorporation of temporal dynamics enabled superior predictive performance and provided a deeper understanding of EEG changes associated with delirium.

Nevertheless, although this model demonstrates strong predictive performance in distinguishing pre- and post-diagnosis EEG states, its primary utility lies in monitoring temporal changes in patients already diagnosed with delirium. Its focus complements the primary models developed in this study, which aimed to predict the risk of delirium at ICU admission.

A nomogram was developed specifically to illustrate the importance of these EEG features recorded before delirium onset, highlighting their role in distinguishing between EEG recordings before and after delirium diagnosis (Fig. 4).

To use it, each EEG feature is assigned a value based on its presence or level, with blue markers indicating the values or categories associated with delirium development in this study cohort. For example, if a patient has absent PDR (0), absent predominant background frequency of 8 to 13 Hz (0), absent AP gradient (0), absent continuous EEG (0), and predominant background frequency of 4 to 7 Hz (1), the nomogram predicts an approximate 96% probability that these EEG findings frequently precede delirium diagnosis, indicating a higher likelihood.

Overall, this pre- and post-diagnosis model adds a temporal dimension and is adaptable to changes in patient status. Integrating the nomogram into clinical practice may facilitate better monitoring and intervention strategies for delirium by helping clinicians understand and quantify changes in EEG features over time, thereby enhancing the assessment of delirium progression.

## Discussion

In this study, various EEG-based ML models were evaluated to improve the knowledge and predictive accuracy of delirium in ICU patients with COVID-19. The developed models included those that analyzed individual EEG features, combined multiple EEG features, incorporated comprehensive EEG variables, and integrated EEG, demographic, clinical, and laboratory variables. Additionally, a model was devised to assess EEG changes before and after delirium diagnosis, offering a dynamic perspective on the condition.

### Key findings

The most reliable EEG predictors of delirium, consistently ranked highly across most models, include the following: the presence of a predominant background frequency of 4 to 7 Hz (increased theta activity) or less than 4 Hz (increased delta activity), RPPs, and low voltage; and the absence of PDR, a predominant background frequency of 8 to 13 Hz (reduction in alpha activity), state changes, a continuous EEG, and the AP gradient. These features are uncommon in awake, healthy adults [95], and typically indicate altered brain states and neuronal instability. Furthermore, globally, these findings suggest a breakdown in cognitive function in patients with delirium, which is consistent with a model previously proposed [94].

Several of these findings also align with early descriptions of EEG slowing as a hallmark of delirium [45], and with the existing literature, which consistently identifies increased theta and delta activity, along with a reduction in alpha activity, as key EEG indicators during both pre-delirium and delirium phases [40, 41, 47, 82, 83, 86, 94–102]. The observed diffuse slowing of EEG and reduction in alpha activity before delirium are meaningful, as they indicate a shift from normal cortical functioning, which is often associated with dominant alpha rhythms during relaxed wakefulness, toward the disorganized and impaired state typical of delirium with slower EEG frequencies [83, 98]. Moreover, as demonstrated previously, reduced alpha power is associated with increased vulnerability to delirium [103], and changes in the alpha band EEG activity are closely linked to acute alterations in arousal and cognitive function [104]. Similarly, lower-frequency EEG backgrounds are also strongly associated with disruptions in cortical network synchronization, which reflects impaired cognitive processing and broader brain network disconnection in delirious patients, even when arousal levels appear normal [47, 86]. The presence of delta activity, which is considered a pathological finding in adult awake EEGs and a cardinal EEG feature of encephalopathy [46], and theta rhythms, which are commonly associated with decreased cognitive performance [105, 106], support this idea of widespread cortical dysregulation. These EEG changes likely reflect the evolving progression of a state of diminished cortical efficiency and may result from well-established disruptions in large-scale neuronal networks in the brain [17, 107], contributing to cognitive dysfunction and core delirium features, such as fluctuating disturbances in attention and consciousness [64]. Overall, the consistent role of lower-frequency EEG variables across all models, in the present study, reinforces their value as biomarkers for predicting the onset of delirium, supporting the well-documented shift toward lower-frequency activity as a defining aspect of the delirium EEG profile.

Similarly, the absence of PDR emerged as a frequent EEG abnormality prior to delirium development, as identified in the combined model analyzing literature-supported EEG variables and the model based on temporal EEG changes. This finding is consistent with those of previous studies [86, 91]. PDR, an alpha rhythm in the 8–13 Hz range observed in the occipital regions of the brain upon eye closure, is common in healthy adults [83] and serves as a marker of normal cortical function and thalamocortical connectivity [91]. Its absence, reduction, or significant alteration, such as a shift toward slower theta or delta frequencies, has been associated with various forms of encephalopathy, including those leading to delirium [36, 83, 86, 108]. In critically ill patients, the absence of PDR, along with the emergence of slower EEG frequencies, is indicative of more severe neurological impairment and is considered a critical marker for assessing the severity and progression of encephalopathic conditions, similar to delirium [108]. Therefore, in the context of ICU patients with severe infections, such as SARS-CoV-2, in whom neurological symptoms and complications are increasingly reported [109–111], the absence of PDR may serve as a crucial biomarker for early delirium detection.

Regarding the association between RPPs and delirium, RPPs are common in ICU patients and reflect abnormal but rhythmic cortical activity, which is frequently associated with impaired neural synchronization and indicative of significant acute or subacute brain impairment [67, 112, 113]. Previous studies have highlighted the potential role of RPPs as markers of cortical instability in critically ill populations. These patterns, including generalized PDs, were found to be significantly more common in ICU patients with delirium (43% vs. 22%, *p* = 0.03) and were associated with an increased risk of delirium, even in the absence of seizures [41]. Furthermore, the inclusion of PDs in predictive EEG models has been demonstrated to enhance sensitivity, particularly when combined with more common features such as generalized slowing and absent reactivity [86]. Collectively, the evidence suggests that, although RPPs are not specific to delirium, their increased prevalence in these patients highlights their utility as complementary markers of cortical dysfunction and their potential to improve the accuracy and reliability of delirium prediction and monitoring in ICU settings.

State changes, defined as transitions of at least two sustained background EEG patterns related to alertness or stimulation, reflect the brain’s ability to respond to stimuli [67] and depend on the integrity of the cortical and thalamocortical networks, which are critical for regulating arousal [114] and enabling the dynamic synchronization needed for transitions between brain states [115]. In this study, their absence was associated with delirium, suggesting impaired cortical adaptability and failure of the neural circuits required for state transitions. This aligns with previous findings that the absence of a dominant awake-state EEG rhythm [116] and reduced alpha-band connectivity [117–119] are prevalent in delirium, indicating an inability to maintain or transition between organized brain states. Both phenomena reflect disruptions in cortical circuit integrity, and because state changes depend on the coordinated activity of these circuits, their absence in delirium may reveal dysfunction in the same underlying mechanisms, with evidence suggesting that connectivity disruptions contribute to impaired consciousness and attention [119]. In addition to the absence of state changes, the lack of continuous EEG activity and AP gradient also emerged as important predictors of delirium. Continuous EEG refers to a stable, ongoing pattern of brain activity typical of normal brain function [95]. Previously, the absence of continuous EEG activity has been observed in delirium [41, 47], suggesting a breakdown in the brain’s adaptive networks, necessary for cognitive function and attention, consistent with the cognitive impairments seen in delirium patients [40, 119, 120]. Regarding the AP gradient, which is typically present in healthy individuals, it reflects organized neural activity, with faster frequencies dominating the anterior regions and slower ones in the posterior regions [67]. In delirium, EEG patterns often display generalized delta and theta activity and a poorly organized background rhythm, which collectively contribute to the absence of the AP gradient [86, 89, 108]. This is further supported by reports of a loss of posterior-to-anterior directionality in the alpha band, reflecting disrupted cortical connectivity in delirium[40, 119]. These three findings—absent state changes, absent continuous EEG, and absent AP gradient—could be interconnected and represent distinct but related aspects of widespread cortical dysfunction in delirium. State changes rely on the brain’s ability to transition dynamically between functional states, continuous EEG activity reflects the maintenance of stable background activity, and the AP gradient indicates the spatial organization of neural networks. The combined absence of these features suggests severe disruptions in the cortical and thalamocortical networks, impairing brain adaptability, synchronization, and connectivity. Additionally, these shared mechanisms are likely central to the impaired consciousness and attention characteristic of delirium. Further research is needed to explore how these EEG abnormalities interact and whether combining them can improve prediction and management, particularly in ICU settings.

Finally, a transition to EEG background with low voltages (< 20 µV) proved to be a highly specific indicator of delirium, although its standalone diagnostic utility is limited by low sensitivity. In this study, low voltage, as an individual model, demonstrated a specificity of 0.949 in predicting delirium when present, but a sensitivity of only 0.129, failing to identify most delirium cases. These findings agree with prior studies, including one that reported low voltage or generalized attenuation as highly specific for diagnosing delirium, with a specificity of 98.7%, although its sensitivity was much lower at 1.7% [86]. Regarding the present study, in the cohort used for all models except the one focusing on temporal changes, only one non-delirium patient had an EEG with a suppressed voltage (< 10 µV), and one non-delirium patient had a low voltage EEG (10 to 20 µV), compared with four delirium patients with low voltage (10 to 20 µV). These findings suggest that less severe reductions in EEG amplitude, such as low voltage (10 to 20 µV), appeared to be more indicative of delirium. In support of this, a recent study found that low voltage (< 20 µv) was more common in patients with delirium (4.1%) than in non-delirium patients (1.5%). In contrast, extreme low voltage or electrocerebral silence did not show a significant difference between the two groups of patients [89]. These results highlight the utility of low voltage as part of a broader diagnostic framework. Although its rarity limits its utility as an isolated feature, its high specificity makes it a valuable marker when combined with other EEG variables, offering enhanced predictive power in identifying delirium in critically ill patients.

Other well-known EEG abnormalities associated with delirium, such as the absence of reactivity or occurrence of SEDs, did not emerge as meaningful predictors in this study. This suggests that although these EEG abnormalities are commonly observed in patients with ICU delirium, they may not have a strong predictive value for the specific population assessed in this context.

Concerning the ML models focused on predicting delirium using only EEG variables, the best-performing model was the one that integrated multiple EEG features (comprehensive EEG model), rather than relying on a single feature or solely combining literature-supported EEG features. This model achieved an AUC of 0.733, demonstrating a moderate ability to predict delirium. Compared with the other EEG models developed, the comprehensive EEG model also provided a more balanced performance, with a sensitivity of 0.645 and specificity of 0.692, suggesting that combining EEG biomarkers better captures the complex neural dynamics associated with delirium. Nevertheless, the challenge of simultaneously achieving high sensitivity and specificity persisted across all models relying exclusively on EEG variables. To the best of the authors’ knowledge, there are no clearly documented explanations for the modest sensitivity and specificity values observed in these EEG-based ML models. However, it is believed that this ongoing issue may be attributed not only to the limitations of visual EEG inspection and the need for more sophisticated feature extraction and processing methods, but also to the inherent variability of EEG signals, patient-specific variability, the effects of medications and sedation, the multifactorial and heterogeneous nature of delirium, and the temporal dynamics of the condition. Addressing these factors could enhance the model’s ability to both balance and elevate these metrics more effectively.

Direct comparisons between this study and others are challenging due to differences in methodology, patient populations, and the EEG data collected. After extensive research, no other study has been found to use the unique methodology employed here, which includes routine EEG with 21 channels, visual EEG analysis, a specific ML approach, and ICU patients. Furthermore, studies with similar aims have reported variable findings. For instance, a ML model developed to predict postoperative delirium in older patients using intraoperative frontal EEG signatures achieved an AUC of 0.770, which is comparable to the AUC of 0.733 reported in this study’s comprehensive EEG model [59]. The similarities in AUC suggest that, despite different patient populations and specific methodologies, both studies faced similar challenges in predicting delirium using EEG data and ML techniques. In contrast, a higher AUC of 0.93 was reported for a ML model that also predicts postoperative delirium using EEG data obtained from a specialized portable EEG device in patients undergoing cardiovascular surgeries [60]. Several factors likely contributed to this outcome, e.g., the use of preoperative EEG data in a controlled setting, a focus on gamma band activity, and the implementation of a robust ML model (Extra Trees Classifier). Additionally, the relatively homogeneous patient population undergoing similar types of surgery may have reduced variability, thereby enhancing the model’s ability to detect consistent EEG patterns related to delirium risk. More recently, a study using deep learning models with Vision Transformers achieved over 97% accuracy in predicting delirium using limited lead EEG data in critically ill ICU patients [48]. This high accuracy may reflect the advanced capabilities of Vision Transformers in handling complex EEG data and the data augmentation techniques used to enhance the training process. However, it is important to note that the methodology and patient population in that study differ from the present one, which exclusively includes patients with COVID-19 admitted to the ICU, who present unique challenges and variability that may impact predictive accuracy. Despite the methodological differences, these findings highlight the consistency of EEG-based approaches across diverse contexts and patient groups. Nevertheless, although EEG changes can be associated with delirium, careful interpretation is necessary because their predictive accuracy may be limited when used independently, as demonstrated by the individual EEG feature models developed in the present study, and collectively. Standardized and comparable studies are urgently needed to better understand the true efficacy of EEG in predicting delirium across diverse patient populations and clinical settings.

Incorporating demographic, clinical, and laboratory variables (e.g., sex, COVID-19 vaccination status, and administration of sodium chloride) alongside EEG features resulted in an AUC of 0.825. This value surpasses the AUC of the best model developed using only EEG features (e.g., the comprehensive EEG model with an AUC of 0.733), although it still falls short of achieving perfect predictive accuracy. Additionally, the model achieved a sensitivity of 0.613 and a specificity of 0.795, demonstrating that including demographic, clinical and/or laboratory factors alongside EEG biomarkers enhances the model’s ability to predict delirium, particularly by improving specificity while maintaining a reasonable level of sensitivity. The slight improvement in specificity indicates that integrating the clinical context with EEG data helps reduce the rate of false positives, making the model more reliable in real-world ICU settings where comprehensive patient data are available. Similarly, integrating intraoperative frontal EEG signatures with clinical parameters such as age, American Society of Anesthesiology score, length of operation, and medication used for anesthesia improved the prediction of postoperative delirium [59]. This combined model achieved an overall AUC of 0.77, with the highest AUC of 0.80 obtained for patients receiving Sevoflurane, demonstrating that the inclusion of both EEG data and clinical parameters enhances predictive accuracy compared with models using only EEG features. These findings suggest that a holistic approach combining EEG features with clinical assessment is valuable for accurate prediction. Interestingly, although certain EEG features played a role in the model’s development (i.e., predominant background frequency of 4 to 7 Hz and absence of state changes), the variables sex, COVID-19 vaccination status, and administration of sodium chloride also emerged as top-ranked variables, highlighting their significant influence on delirium risk. Indeed, male sex has been associated with an increased vulnerability to delirium [82, 83, 121], as well as the lack of COVID-19 vaccination [84], with unvaccinated individuals being more susceptible to severe disease progression and inflammation. Additionally, the administration of sodium chloride, which can lead to electrolyte imbalances, such as hyperchloremia [122], may increase the risk of delirium by disrupting neural activity and impairing brain function in critically ill patients [123, 124].

The model assessing EEG changes before and after delirium diagnosis demonstrated high accuracy (0.818) and AUC (0.950), indicating a strong capability to detect temporal EEG changes associated with delirium onset. This model identified key EEG changes before delirium diagnosis, such as the absence of PDR, an AP gradient, and a continuous pattern of EEG, and the increase in theta activity along with the reduction in alpha activity. In comparison, a study analyzing EEGs during delirium and on control days (at least one-week post-transfer or discharge, without delirium) also found notable differences [125]. During delirium, there was a pronounced increase in slow-wave activity and a marked reduction in alpha waves, contrasting with the stable EEG patterns on the control days. These findings emphasize the distinct EEG signatures of delirium, validating the use of multiple EEGs for monitoring and detection [125]. Similar EEG changes have also been observed in neurodegenerative diseases, where they serve as key indicators of cognitive vulnerability. This parallel suggests that EEG alterations may reflect a broader risk of cognitive dysfunction across multiple conditions, positioning EEG as a valuable tool for identifying high-risk patients who may benefit from further diagnostic evaluation and early intervention [44]. Due to the lack of recent studies using ML models and repeated EEG recordings, Matsushima et al.’s (1997) study is referenced, despite not employing advanced methodologies. Nevertheless, these findings still underline the dynamic nature of EEG alterations in delirium, demonstrating that continuous EEG data can facilitate early detection and timely intervention, thus supporting the broader use of EEG-based prediction models in clinical settings. Temporal EEG analysis not only highlights the dynamic nature of these alterations but also provides actionable insights into the progression and recovery of delirium. For example, the re-emergence of PDR or AP gradients observed in the present study could serve as reliable indicators of recovery, helping clinicians tailor treatment plans, such as tapering sedation, when EEG patterns normalize. These dynamic assessments are particularly valuable in ICU settings, where the clinical symptoms of delirium may fluctuate or overlap with the effects of sedation. By providing a more nuanced understanding of these patterns, temporal EEG data can support evidence-based monitoring and intervention approaches.

### Clinical relevance

EEG-based ML models have the potential to predict delirium in ICU settings. However, their practical application is hindered by the moderate AUC, specificity, and sensitivity. In the ICU, missing delirium (false negatives) poses significant risks, including delayed interventions and worse outcomes, whereas false positives may lead to unnecessary treatments. These risks highlight the need for the cautious integration of these models into clinical practice, emphasizing their role as part of a comprehensive diagnostic framework. By combining EEG findings with clinical assessments and other diagnostic tools—such as temporal EEG monitoring and key risk factors like sex or metabolic imbalances—it is possible to enhance accuracy and facilitate early detection. This integrated approach can improve patient outcomes while minimizing the risk of misclassification.

### Limitations

Several limitations of this study must be acknowledged. First, the relatively small sample size may limit the generalizability of the findings and increase the risk of overfitting. To mitigate this, cross-validation techniques were employed during model development, reducing the likelihood of this issue and improving the robustness. However, larger studies are needed to confirm these findings and ensure broader applicability. Thus, these results should be interpreted with caution and should not be overstated without further evidence. Second, the moderate specificity and sensitivity of most of the models indicate that further refinement is necessary. In the ICU setting, where misclassified delirium cases (false negatives) can have severe consequences, sensitivity is particularly critical. Improving sensitivity without significantly compromising the specificity should be the focus of future iterations of these models. Third, the EEG signals in this study were evaluated through visual inspection rather than computational processing. Although this approach aligns with current clinical practices, it may overlook subtle patterns that are detectable through advanced signal processing techniques. Using bio-signal data from quantitative EEG could pave the way for developing more advanced algorithms to predict delirium in ICUs [126], a direction that the authors of this study are actively exploring to enhance clinical decision-making. Fourth, external validation was not performed due to the limited availability of independent datasets containing both EEG recordings and corresponding clinical data from ICU patients with COVID-19. This limitation reflects the challenges of accessing standardized, high-quality datasets across institutions, particularly in critically ill populations. Nonetheless, cross-validation helped mitigate the risk of overfitting and provided a reliable estimate of the models’ performance. Future studies should aim to validate these findings using external cohorts to ensure wider applicability and generalizability. Fifth, the effects of sedation and other medications on EEG patterns were not fully accounted for in this study. Two chi-square tests were conducted to evaluate the association between sedation during EEG and the presence of suppressed or low voltage: one including all patients and another restricted to those with delirium. Neither test revealed a significant relationship (*p* = 0.656 and *p* = 0.901, respectively); however, sedation may still influence other EEG features relevant to delirium prediction. These medications can obscure or mimic features associated with delirium, potentially affecting the accuracy of EEG-based predictions. This limitation underscores the need for future studies to carefully control for medication effects, ensuring that the observed EEG patterns are directly linked to delirium rather than confounding pharmacological influences. Nonetheless, no similar studies have used routine 21-channel EEGs in ICU settings combined with ML models, highlighting the uniqueness of this approach. This novelty, however, also limits the ability to directly compare the findings with the existing literature, making it difficult to contextualize the findings within broader research frameworks. Finally, implementing these ML models in clinical practice requires careful consideration of practical aspects such as the availability of EEG equipment, the training of clinical staff, and the integration of these tools into existing ICU workflows. Additionally, the cost-effectiveness of using EEG-based models for delirium prediction and management should also be assessed.

### Future research

Future research should focus on refining ML models to enhance AUC, specificity, and sensitivity, particularly by incorporating larger datasets, optimizing feature extraction, and exploring the integration of additional clinical variables, such as metabolic or inflammatory markers. Leveraging advanced EEG features derived from quantitative analyses could further improve model performance, while longitudinal studies are essential to better understand the temporal dynamics of EEG changes in delirium and their relationship to clinical outcomes. These efforts could help identify early biomarkers of delirium progression, enabling timely and targeted interventions. Simultaneously, developing real-time predictive models that integrate dynamic EEG data into ICU workflows could enhance their practical applicability and support personalized patient care. Additionally, exploring multimodal approaches—combining EEG with neuroimaging, autonomic measures, or other biomarkers—could provide a more comprehensive framework for delirium detection and management. Future models should also consider the impact of sedation on EEG patterns. Stratifying patients according to sedation levels, types, or timing, or incorporating sedation-adjusted EEG biomarkers into models could further enhance prediction accuracy in ICU settings.

## Conclusions

This study demonstrated the potential of EEG-based ML models to predict delirium in critically ill patients by identifying specific markers of cortical dysfunction, such as increased theta activity. The best-performing model based on EEG features achieved an AUC of 0.733, with a sensitivity of 0.645 and a specificity of 0.692, reflecting its moderate ability to differentiate between delirium and non-delirium cases. While promising, these performance metrics emphasize the need for further refinement through the integration of additional clinical and biological data, as well as validation in a broader patient population. Incorporating these EEG-based tools into a comprehensive diagnostic framework could facilitate earlier detection, personalized interventions, and improved outcomes in ICU settings. Furthermore, this study contributes to the growing field of precision medicine, offering a foundation for advancing data-driven care for critically ill patients.

## Supplementary Information

Below is the link to the electronic supplementary material.

Supplementary file1 (DOCX 800 KB)

## Data Availability

The datasets generated and/or analyzed during the current study are available from the corresponding author on reasonable request.
