# Supplementary material for: Predicting delirium in critically Ill COVID-19 patients using EEG-derived data: a machine learning approach
Source: GeroScience. 2025 Jul 23;48(2):2617–45. doi: 10.1007/s11357-025-01809-0 (PMC12972162; doi:10.1007/s11357-025-01809-0)
Supplement: Supplementary file 1 — Supplementary file1 (DOCX 800 KB) [file 11357_2025_1809_MOESM1_ESM.docx]

**SUPPLEMENTARY MATERIALS**

**Predicting delirium in critically Ill COVID-19 patients using EEG-derived data: a machine learning approach**

**Ana Viegas^1,2,3,4,5^** | **Cristiana P. Von Rekowski^1,2,6^** | **Rúben Araújo^1,2,6^** | **Luís Ramalhete^1,7,8^** | **Inês Menezes Cordeiro^5,9^** | **Manuel Manita^5,9^** | **Miguel Viana-Baptista^1,11,12^** | **Paula Macedo^1,8^** | **Luís Bento**[**^1,2,10^**](#_bookmark7)

1. NMS – NOVA Medical School, FCM – Faculdade de Ciências Médicas, Universidade NOVA de Lisboa, Campo dos Mártires da Pátria 130, 1169-056 Lisbon, Portugal
2. CHRC – Comprehensive Health Research Centre, Universidade NOVA de Lisboa, Campo dos Mártires da Pátria 130, 1150-082 Lisbon, Portugal
3. ESTeSL – Escola Superior de Tecnologia da Saúde de Lisboa, Instituto Politécnico de Lisboa, Avenida D. João II, Lote 4.69.01, Parque das Nações, 1990-096 Lisbon, Portugal
4. H&TRC – Health & Technology Research Center, ESTeSL – Escola Superior de Tecnologia da Saúde de Lisboa, Instituto Politécnico de Lisboa, Avenida D. João II, Lote 4.69.01, Parque das Nações, 1990-096 Lisbon, Portugal
5. Neurosciences Area, Clinical Neurophysiology Unit, ULSSJ – Unidade Local de Saúde São José, Rua José António Serrano, 1150-199 Lisbon, Portugal
6. ISEL– Instituto Superior de Engenharia de Lisboa, Instituto Politécnico de Lisboa, Rua Conselheiro Emídio Navarro 1, 1959-007 Lisbon, Portugal
7. Blood and Transplantation Center of Lisbon, Instituto Português do Sangue e da Transplantação, Avenida Miguel Bombarda 6, 1000-208 Lisbon, Portugal
8. iNOVA4Health – Advancing Precision Medicine, NOVA Medical School, FCM – Faculdade de Ciências Médicas, Universidade NOVA de Lisboa, Campo dos Mártires da Pátria 130, 1169-056 Lisbon, Portugal
9. Neurology Department, ULSSJ – Unidade Local de Saúde São José, Rua José António Serrano, 1150-199 Lisbon, Portugal
10. Intensive Care Department, ULSSJ – Unidade Local de Saúde São José, Rua José António Serrano, 1150-199 Lisbon, Portugal
11. Neurology Department, ULSLO – Unidade Local de Saúde de Lisboa Ocidental, Rua da Junqueira 126, 1349-019 Lisbon, Portugal
12. CCAL – Centro Clínico Académico de Lisboa, NOVA Medical School, FCM – Faculdade de Ciências Médicas, Universidade NOVA de Lisboa, Campo dos Mártires da Pátria 130, 1169-056 Lisbon, Portugal

**Corresponding author:**

- Name: Ana Isabel Loureiro Viegas
- Address: Nova Medical School – NOVA University Lisbon, [Campo dos Mártires da Pátria 130, 1169-056 Lisboa](https://www.google.com/maps/place/data=!4m2!3m1!1s0xd19347a6b91c653:0x34754c7fa7c00c26?sa=X&ved=1t:8290&ictx=111)
- Email: [a2020449@nms.unl.pt](mailto:a2020449@nms.unl.pt)

**Supplementary materials:**

**Table S1:** List of the Electroencephalogram (EEG) features collected for this study and their corresponding categorization. *Abbreviations:* AP, Anterior-posterior; BIRDs, Brief Potentially Ictal Rhythmic Discharges; CAPE, Cyclic Alternating Pattern of Encephalopathy; EEG, Electroencephalography; ICC, Ictal-interictal Continuum; PDA, Polymorphic Delta Activity; PDR, Posterior Dominant Rhythm; RPPs, Rhythmic or Periodic Patterns; SE, Status Epilepticus; SEDs, Sporadic Epileptiform Discharges; SIRPIDs, Stimulus-induced Rhythmic, Periodic, or Ictal-appearing Discharges.

| EEG feature | Categorization |
| --- | --- |
| Posterior Dominant Rhythm (PDR) | 1 – Present; 0 – Absent |
| Predominant background frequency:   - Less than 4 Hz. - 4 to 7 Hz. - 8 to 13 Hz. - 14 to 30 Hz. | 1 – Present; 0 – Absent |
| Continuity:   - Continuous. - Nearly Continuous. - Discontinuous. - Burst-suppression/Burst-attenuation. - Suppression. | 1 – Present; 0 – Absent |
| Reactivity:   - Reactive. - Unreactive. - Stimulus-induced Rhythmic, Periodic, or Ictal-appearing Discharges (SIRPIDs) only. - Unclear. - Unknown. | 1 – Present; 0 – Absent |
| State changes | 1 – Present; 0 – Absent |
| Cyclic Alternating Pattern of Encephalopathy (CAPE) | 1 – Present; 0 – Absent |
| Voltage:   - Normal voltage. - Suppressed or Low Voltage. - High Voltage. | 1 – Present; 0 – Absent |
| Anterior-Posterior (AP) Gradient:   - Present. - Absent. - Reverse. | 1 – Present; 0 – Absent |
| Breach effect | 1 – Present; 0 – Absent |
| Sporadic Epileptiform Discharges (SEDs) | 1 – Present; 0 – Absent |
| Rhythmic and Periodic Patterns (RPPs) | 1 – Present; 0 – Absent |
| Electrographic or electroclinical seizures | 1 – Present; 0 – Absent |
| Brief Potentially Ictal Rhythmic Discharges (BIRDs) | 1 – Present; 0 – Absent |
| Ictal-Interictal Continuum (ICC) | 1 – Present; 0 – Absent |
| Frontal abnormalities | 1 – Present; 0 – Absent |
| Status Epilepticus (SE) | 1 – Present; 0 – Absent |
| Polymorphic Delta Activity (PDA) | 1 – Present; 0 – Absent |

**Table S2:** Comparison of ICU admission reasons between 31 patients with delirium and 39 without, with *p*-values from Fisher’s exact test or the chi-square test. *Abbreviations:* COVID-19, Coronavirus Disease 2019; ICU, Intensive Care Unit.

|  | Delirium patients  (*n* = 31) | Non-delirium patients  (*n* = 39) | *p*-value | Statistic test |
| --- | --- | --- | --- | --- |
| Acute respiratory failure due to COVID-19 (n, %) | 29 (93.5%) | 34 (87.2%) | 0.936 | Chi-square test |
| Urgent surgery (n, %) | 1 (3.2%) | 0 (0.0%) | 0.451 | Fisher’s exact test |
| Septic shock (n, %) | 0 (0.0%) | 1 (2.6%) | 1.000 | Fisher’s exact test |
| Renal insufficiency (n, %) | 0 (0.0%) | 1 (2.6%) | 1.000 | Fisher’s exact test |
| Guillain-Barré syndrome (n, %) | 0 (0.0%) | 1 (2.6%) | 1.000 | Fisher’s exact test |
| Acute myocardial infarction (n, %) | 0 (0.0%) | 1 (2.6%) | 1.000 | Fisher’s exact test |
| Heart rhythm changes (n, %) | 0 (0.0%) | 1 (2.6%) | 1.000 | Fisher’s exact test |
| Others | 1 (3.2%) | 0 (0.0%) | 1.000 | Fisher’s exact test |

**Table S3:** Comparison of the number of patients admitted during each Portuguese COVID-19 infection wave between 31 patients with delirium and 39 without, with *p*-values from Fisher’s exact test or the chi-square test. *Abbreviations:* COVID-19, Coronavirus Disease 2019.

|  | Delirium patients  (*n* = 31) | Non-delirium patients  (*n* = 39) | *p*-value | Statistic test |
| --- | --- | --- | --- | --- |
| Wave 1 (n, %) | 4 (12.9%) | 1 (2.6%) | 0.163 | Fisher's exact test |
| Wave 2 (n, %) | 0 (0.0%) | 0 (0.0%) | Not applicable | Constant data |
| Wave 3 (n, %) | 6 (19.4%) | 2 (5.1%) | 0.127 | Fisher's exact test |
| Wave 4 (n, %) | 16 (51.6%) | 24 (61.5%) | 0.405 | Chi-square test |
| Wave 5 (n, %) | 4 (12.9%) | 11 (28.2%) | 0.150 | Fisher's exact test |
| Wave 6 (n, %) | 1 (3.2%) | 1 (2.6%) | 1.000 | Fisher's exact test |

**Table S4:** Confusion matrix for the Logistic Regression combined delirium prediction model using literature-supported EEG variables. *Abbreviations*: EEG, Electroencephalography.

|  |  | Predicted | |  |
| --- | --- | --- | --- | --- |
| Real |  | No delirium | Delirium | **Σ** |
|  | No delirium | 65.9% (**29**) | 38.5% (**10**) | 39 |
|  | Delirium | 34.1% (**15**) | 61.5% (**16**) | 31 |
|  | **Σ** | 44 | 26 | 70 |

**Table S5:** Confusion matrix for the Naïve Bayes delirium prediction model using comprehensive EEG variables. *Abbreviations*: EEG, Electroencephalography.

|  |  | Predicted | |  |
| --- | --- | --- | --- | --- |
| Real |  | No delirium | Delirium | **Σ** |
|  | No delirium | 71.1% (**27**) | 37.5% (**12**) | 39 |
|  | Delirium | 28.9% (**11**) | 62.5% (**20**) | 31 |
|  | **Σ** | 38 | 32 | 70 |

**Table S6:** Final set of demographic, clinical, and laboratory features used in developing the delirium prediction model based on EEG, demographic, clinical, and laboratory variables, along with their corresponding categorizations. *Abbreviations:* COVID-19, Coronavirus Disease 2019; ECMO, Extracorporeal Membrane Oxygenation; ICU, Intensive Care Unit; IMV, Invasive Mechanical Ventilation.​

| Feature | Categorization |
| --- | --- |
| Reason for ICU admission | 1 – Acute respiratory failure due to COVID-19  2 – Urgent surgery  3 – Acute myocardial infarction  4 – Stroke  5 – Septic chock  6 – Heart rhythm changes  7 – Guillain-Barré syndrome  8 – Renal insufficiency  9 – Other reason |
| Age | Numerical (years) |
| Alanine Aminotransferase (maximum) | Numerical (U/L) |
| Alanine Aminotransferase (minimum) | Numerical (U/L) |
| Alkaline Phosphatase (maximum) | Numerical (U/L) |
| Alkaline Phosphatase (minimum) | Numerical (U/L) |
| Alprazolam | 1 – Present; 0 – Absent |
| Amoxicillin + Clavulanic Acid | 1 – Present; 0 – Absent |
| Arterial Blood Gas \| Lactate (maximum) | Numerical (mmol/L) |
| Arterial Blood Gas \| Lactate (minimum) | Numerical (mmol/L) |
| Arterial Blood Gas \| pCO2 (maximum) | Numerical (mmHg) |
| Arterial Blood Gas \| pCO2 (minimum) | Numerical (mmHg) |
| Arterial Blood Gas \| pO2 (maximum) | Numerical (mmHg) |
| Arterial Blood Gas \| pO2 (minimum) | Numerical (mmHg) |
| Arterial Hypertension | 1 – Present; 0 – Absent |
| Aspartate Aminotransferase (maximum) | Numerical (U/L) |
| Aspartate Aminotransferase (minimum) | Numerical (U/L) |
| Atorvastatin | 1 – Present; 0 – Absent |
| Azithromycin | 1 – Present; 0 – Absent |
| Beclomethasone | 1 – Present; 0 – Absent |
| Bisacodyl | 1 – Present; 0 – Absent |
| Budesonide | 1 – Present; 0 – Absent |
| C-Reactive Protein (maximum) | Numerical (mg/L) |
| C-Reactive Protein (minimum) | Numerical (mg/L) |
| Calcium (maximum) | Numerical (mg/dL) |
| Calcium (minimum) | Numerical (mg/dL) |
| Calcium Gluconate (therapeutics) | 1 – Present; 0 – Absent |
| Captopril (therapeutics) | 1 – Present; 0 – Absent |
| Ceftriaxone (therapeutics) | 1 – Present; 0 – Absent |
| Chronic Respiratory Disease | 1 – Present; 0 – Absent |
| Complete Blood Count \| Erythrocytes (maximum) | Numerical (10^12/L) |
| Complete Blood Count \| Erythrocytes (minimum) | Numerical (10^12/L) |
| Complete Blood Count \| Hematocrit (maximum) | Numerical (%) |
| Complete Blood Count \| Hematocrit (minimum) | Numerical (%) |
| Complete Blood Count \| Hemoglobin (maximum) | Numerical (g/L) |
| Complete Blood Count \| Hemoglobin (minimum) | Numerical (g/L) |
| Complete Blood Count \| Leukocytes (maximum) | Numerical (10^9/L) |
| Complete Blood Count \| Leukocytes (minimum) | Numerical (10^9/L) |
| Complete Blood Count \| Lymphocytes (maximum) | Numerical (10^9/L) |
| Complete Blood Count \| Lymphocytes (minimum) | Numerical (10^9/L) |
| Complete Blood Count \| Neutrophils (maximum) | Numerical (10^9/L) |
| Complete Blood Count \| Neutrophils (minimum) | Numerical (10^9/L) |
| Complete Blood Count \| Platelets (maximum) | Numerical (10^9/L) |
| Complete Blood Count \| Platelets (minimum) | Numerical (10^9/L) |
| Creatine Kinase (maximum) | Numerical (U/L) |
| Creatine Kinase (minimum) | Numerical (U/L) |
| Creatinine (maximum) | Numerical (mg/dL) |
| Creatinine (minimum) | Numerical (mg/dL) |
| Days between COVID-19 symptom onset and ICU admission | Numerical (days) |
| Dexamethasone (therapeutics) | 1 – Present; 0 – Absent |
| Diabetes | 1 – Present; 0 – Absent |
| Dyslipidemia | 1 – Present; 0 – Absent |
| ECMO on ICU admission | 1 – Present; 0 – Absent |
| Electrolytes (therapeutics) | 1 – Present; 0 – Absent |
| Electrolytes + Glucose | 1 – Present; 0 – Absent |
| Enoxaparin Sodium (therapeutics) | 1 – Present; 0 – Absent |
| Fentanyl (therapeutics) | 1 – Present; 0 – Absent |
| Furosemide (therapeutics) | 1 – Present; 0 – Absent |
| Gamma-Glutamyl Transferase (maximum) | Numerical (U/L) |
| Gamma-Glutamyl Transferase (minimum) | Numerical (U/L) |
| Glucose (therapeutics) | 1 – Present; 0 – Absent |
| Heparin Sodium (therapeutics) | 1 – Present; 0 – Absent |
| High blood pressure | 1 – Present; 0 – Absent |
| IMV on ICU admission | 1 – Present; 0 – Absent |
| Insulin (therapeutics) | 1 – Present; 0 – Absent |
| Ionogram \| Chloride (maximum) | Numerical (mEq/L) |
| Ionogram \| Chloride (minimum) | Numerical (mEq/L) |
| Ionogram \| Potassium (maximum) | Numerical (mEq/L) |
| Ionogram \| Potassium (minimum) | Numerical (mEq/L) |
| Ionogram \| Sodium (maximum) | Numerical (mEq/L) |
| Ionogram \| Sodium (minimum) | Numerical (mEq/L) |
| Ipratropium Bromide (therapeutics) | 1 – Present; 0 – Absent |
| Ischemic Heart Disease | 1 – Present; 0 – Absent |
| Ketamine (therapeutics) | 1 – Present; 0 – Absent |
| Lactate Dehydrogenase (maximum) | Numerical (U/L) |
| Lactate Dehydrogenase (minimum) | Numerical (U/L) |
| Magnesium Metamizole (therapeutics) | 1 – Present; 0 – Absent |
| Methylprednisolone (therapeutics) | 1 – Present; 0 – Absent |
| Metoclopramide (therapeutics) | 1 – Present; 0 – Absent |
| Midazolam (therapeutics) | 1 – Present; 0 – Absent |
| Monopotassium Phosphate (therapeutics) | 1 – Present; 0 – Absent |
| Morphine (therapeutics) | 1 – Present; 0 – Absent |
| Nationality | 1 – Portugal  2 – African countries  3 – Asian countries  4 – Other European countries  5 – South American countries  6 – North American countries |
| Norepinephrine (therapeutics) | 1 – Present; 0 – Absent |
| Obesity | 1 – Present; 0 – Absent |
| Pantoprazole (therapeutics) | 1 – Present; 0 – Absent |
| Paracetamol (therapeutics) | 1 – Present; 0 – Absent |
| Piperacillin + Tazobactam (therapeutics) | 1 – Present; 0 – Absent |
| Procalcitonin (maximum) | Numerical (ng/mL) |
| Procalcitonin (minimum) | Numerical (ng/mL) |
| Propofol (therapeutics) | 1 – Present; 0 – Absent |
| Rocuronium Bromide (therapeutics) | 1 – Present; 0 – Absent |
| Salbutamol (therapeutics) | 1 – Present; 0 – Absent |
| Sennosides A + B (therapeutics) | 1 – Present; 0 – Absent |
| Sex | 1 – Female; 0 – Male |
| Sodium Chloride (therapeutics) | 1 – Present; 0 – Absent |
| Standardized Polymeric Liquid Diet (therapeutics) | 1 – Present; 0 – Absent |
| Thiamine (therapeutics) | 1 – Present; 0 – Absent |
| Total Bilirubin (maximum) | Numerical (mg/dL) |
| Total Bilirubin (minimum) | Numerical (mg/dL) |
| Prothrombin Time (maximum) | Numerical (INR) |
| Prothrombin Time (minimum) | Numerical (INR) |
| Urea (maximum) | Numerical (mg/dL) |
| Urea (minimum) | Numerical (mg/dL) |
| COVID-19 vaccination status | 1 – Present; 0 – Absent |

**Table S7:** Confusion matrix for the Logistic Regression delirium prediction model based on EEG, clinical, and laboratory variables. *Abbreviations*: EEG, Electroencephalography.

|  |  | Predicted | |  |
| --- | --- | --- | --- | --- |
| Real |  | No delirium | Delirium | **Σ** |
|  | No delirium | 72.1% **(31)** | 29.6% **(8)** | 39 |
|  | Delirium | 27.9% **(12)** | 70.4% **(19)** | 31 |
|  | **Σ** | 43 | 27 | 70 |

**Table S8:** Confusion matrix for the Naïve Bayes delirium prediction model based on temporal EEG changes, targeting EEG changes before delirium diagnosis. *Abbreviations*: EEG, Electroencephalography.

|  | |  | Predicted | | |  | |
| --- | --- | --- | --- | --- | --- | --- | --- |
| Real |  | | | After | Before | | **Σ** |
|  | After | | | 81.8% **(9)** | 18.2% **(2)** | | 11 |
|  | Before | | | 18.2% **(2)** | 82.8% **(9)** | | 11 |
|  | **Σ** | | | 11 | 11 | | 22 |


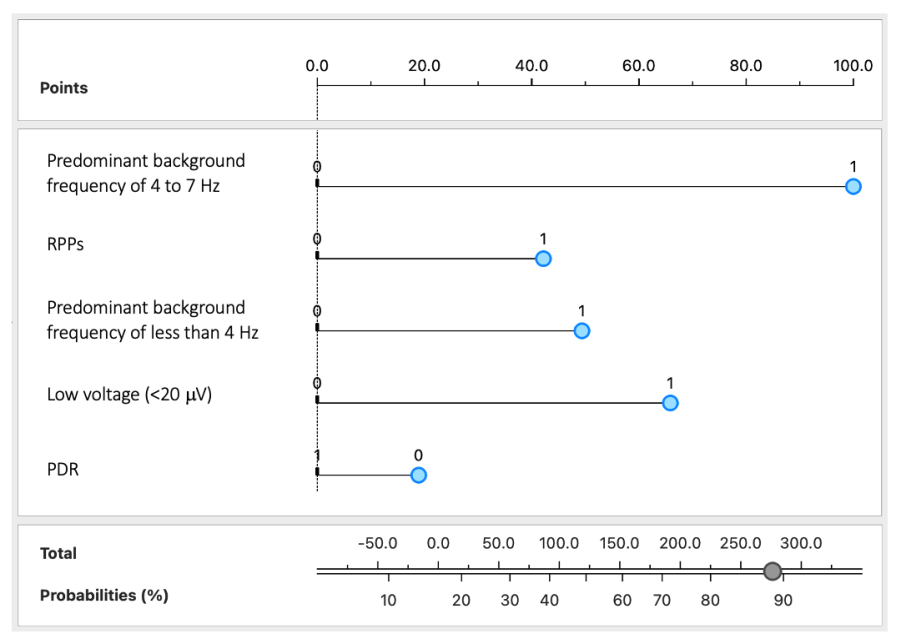


**Figure S1:** Nomogram for the combined delirium prediction model using literature-supported EEG variables. The variables were categorized as follows: predominant background frequency of 4 to 7 Hz, RPPs, predominant background frequency of less than 4 Hz, low voltage (<20 μV), and PDR were indicated by 1 for present and 0 for absent. *Abbreviations:* EEG, Electroencephalography; PDR, Posterior Dominant Rhythm; RPPs, Rhythmic or Periodic Patterns.


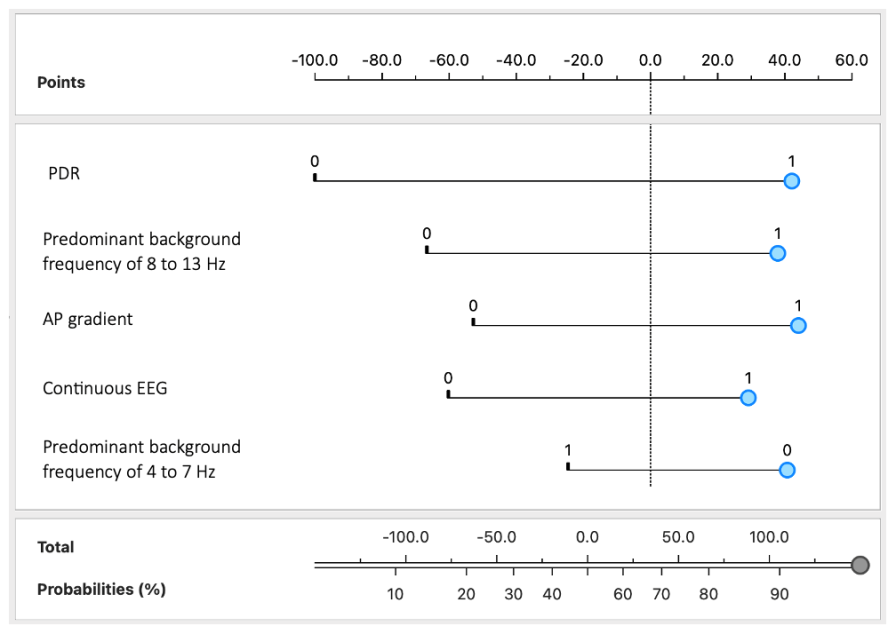


**Figure S2:** Nomogram for the delirium prediction model based on temporal EEG changes targeting EEG changes after delirium diagnosis. The variables were categorized as follows: PDR, predominant background frequency of 8 to 13 Hz, AP gradient, continuous EEG, and predominant background frequency of 4 to 7 Hz were indicated by 1 for present and 0 for absent. *Abbreviations:* AP, Anterior-posterior; EEG, Electroencephalography; PDR, Posterior Dominant Rhythm.
